# Inherited Inflammatory Response Genes Are Associated with B-Cell Non-Hodgkin’s Lymphoma Risk and Survival

**DOI:** 10.1371/journal.pone.0139329

**Published:** 2015-10-08

**Authors:** Kaspar René Nielsen, Rudi Steffensen, Mette Dahl Bendtsen, Maria Rodrigo-Domingo, John Baech, Thure Mors Haunstrup, Kim Steve Bergkvist, Alexander Schmitz, Julie Stoeveve Boedker, Preben Johansen, Karen Dybkaeær, Martin Boeøgsted, Hans Erik Johnsen

**Affiliations:** 1 Department of Clinical Immunology, Aalborg University Hospital, Aalborg, Denmark; 2 Department of Haematology, Aalborg University Hospital, Aalborg, Denmark; 3 Department of Haematopathology, Aalborg University Hospital, Aalborg, Denmark; 4 Clinical Cancer Research Center, Aalborg University Hospital, Aalborg, Denmark; 5 Department of Clinical Medicine, Aalborg University, Aalborg, Denmark; Cornell University, UNITED STATES

## Abstract

**Background:**

Malignant B-cell clones are affected by both acquired genetic alterations and by inherited genetic variations changing the inflammatory tumour microenvironment.

**Methods:**

We investigated 50 inflammatory response gene polymorphisms in 355 B-cell non-Hodgkin’s lymphoma (B-NHL) samples encompassing 216 diffuse large B cell lymphoma (DLBCL) and 139 follicular lymphoma (FL) and 307 controls. The effect of single genes and haplotypes were investigated and gene-expression analysis was applied for selected genes. Since interaction between risk genes can have a large impact on phenotype, two-way gene-gene interaction analysis was included.

**Results:**

We found inherited SNPs in genes critical for inflammatory pathways; *TLR9*, *IL4*, *TAP2*, *IL2RA*, *FCGR2A*, *TNFA*, *IL10RB*, *GALNT12*, *IL12A* and *IL1B* were significantly associated with disease risk and *SELE*, *IL1RN*, *TNFA*, *TAP2*, *MBL2*, *IL5*, *CX3CR1*, *CHI3L1* and *IL12A* were, associated with overall survival (OS) in specific diagnostic entities of B-NHL. We discovered noteworthy interactions between DLBCL risk alleles on *IL10* and *IL4RA* and FL risk alleles on *IL4RA* and *IL4*. In relation to OS, a highly significant interaction was observed in DLBCL for *IL4RA* (rs1805010) * *IL10* (rs1800890) (HR = 0.11 (0.02–0.50)). Finally, we explored the expression of risk genes from the gene-gene interaction analysis in normal B-cell subtypes showing a different expression of *IL4RA*, *IL10*, *IL10RB* genes supporting a pathogenetic effect of these interactions in the germinal center.

**Conclusions:**

The present findings support the importance of inflammatory genes in B-cell lymphomas. We found association between polymorphic sites in inflammatory response genes and risk as well as outcome in B-NHL and suggest an effect of gene-gene interactions during the stepwise oncogenesis.

## Introduction

Normal B-lymphocyte homeostasis requires survival and proliferation signals provided by cells in the lymph node microenvironment. In the case of B-cell non-Hodgkin’s lymphoma (B-NHL) the malignant cells share some similarities with their normal B-cell counterpart linking the immunological inflammatory response to the growth potential of the malignant clone [1–3]. Diffuse large B-cell lymphoma (DLBCL) and follicular lymphoma (FL) accounts for the majority of B-NHL cases [4] and genetic analysis of the tumor microenvironment in these diseases has revealed different gene signatures associated to survival [5–7]. Functional single nucleotide polymorphisms (SNPs) affect the inflammatory microenvironment nesting malignant tumours [8]; however SNPs may also provide a direct effect on the malignant B-cells in the process of tumorigenesis [9]. Different genetic loci are associated with risk or outcome in B-NHL [10–14], amongst them most notably the *IL4RA*, *IL6*, *IL10* and *TNFA* loci [15–21] and recently, genome wide association studies (GWAS) have suggested an effect of a number of other potential loci encoding inflammatory mediators [22,23]. The focus in most of the previous studies has primarily been on single gene effects, however disease susceptibility and prognosis in complex diseases such as B-NHL may not be caused by single genes but by genes interacting [24] as reported by a small number of investigators [10,25–30]. In the present paper we investigate the effect of single gene, haplotype and gene-gene interactions in different histological subtypes of B-NHL. We further suggest, that the biological effect of such interacting inflammatory response genes with germ line polymorphisms is of pathogenetic impact supported by the investigation of “risk genes” expression in normal B-cell compartments and lymphoma tissue [31,32].

## Materials and Methods

### Patients, controls and ethics statement

The current study included 355 B-NHL cases classified according to the World Health Organization (WHO) classification (216 DLBCL and 139 FL). Clinical data was abstracted from the Danish lymphoma database (LYFO) and from medical records. Follow-up information included type of treatment, International Prognostic Index (IPI) score and time to relapse. We included material from 307 healthy blood donors as previously described [33]. Research protocols were approved by the local scientific ethics committee of the North Denmark Region (approval numbers: N-20100059, N-20090018). The use of samples without informed consent was approved by the committee since the result did not result in any interventions and all samples were anonymized and de-identified prior to analysis. For details see S1 Text.

### Sample preparation

Formalin-fixed paraffin-embedded (FFPE) bone-marrow aspirate were used for DNA extraction from all included patients. From controls, DNA were extracted from Ethylenediaminetetraacetic acid (EDTA) stabilised whole blood. For gene-expression analysis, normal lymph node tissue and DLBCL lymphoma cells were processed as previously described [34]. For details see S1 Text.

### Genotyping

Genotyping was performed using a TaqMan OpenArray genotyping system from Applied Biosystems (ABI, Foster City, CA, USA). Seven SNPs were genotyped using custom-designed assays and 43 SNPs were genotyped using predesigned TaqMan SNP assays (see S1 Table for detailed assay information). The arrays were read using the OpenArray NT Imager and the allele calls and scatter plots were generated with the Biotrove OpenArray SNP Genotyping Analysis Software package version 1.0.3. The default threshold for the Quality value was set to 0.95. For details see S1 Text.

### Analysis of gene expression profiles of normal and malignant lymphnodes

DLBCL lymphoma samples and and flow-sorted B-cell populations from Ficoll-purified mononuclear cells from human tonsils were hybridized to Affymetrix GeneChip Human Genome U133 Plus 2.0 Arrays (Affymetrix, Santa Clara, CA) as previously described [3]. The CEL files are deposited in the National Center for Biotechnology Information Gene Expression Omnibus repository (GSE56315)[3]. Gene expression profiles were analyzed using the statistical software system R, version 2.15.3. (http://www.r-project.org) and Bioconductor R-packages [35]. The CEL files were normalized by the just.rma function from the Bioconductor package affy, using customized cdf-files from brainarray [36], which summarized the probesets to ENGS symbols. The ENSG symbols were mapped to the HGNC symbols of interest by the function getBM from the Bioconductor package biomaRt.

### Statistical analysis

Statistical analyses were carried out by the statistical software Stata Version 12.1 (Stata Corporation, College Station, TX, USA) and the statistical software system R, version 2.15.3. (http://www.r-project.org). PHASE 2.1 software (University of Chicago, USA) was used to construct haplotypes. Logistic regression analysis was used to estimate the association between odds of lymphoma and the single genotypes, alleles, haplotypes, and gene-gene interactions, respectively. Survival analysis for 10 year overall survival (OS) was used to analyse the effect of single SNPs, haplotypes, and gene-gene interactions between SNPs corresponding to different genes. Cox proportional hazards models adjusted for sex, age, IPI, and treatment was used for survival analysis. P-values below 0.05 were generally considered statistically significant. The purpose of performing gene-gene interaction analyses was to identify pairs of genes for which the interaction between them was significantly associated with the risk of getting lymphoma (performed by logistic regression) or significantly associated with the survival among lymphoma patients (performed by Cox regression). We performed the gene-gene interaction analyses by comparing the models with and without the interaction term using a likehood ratio test. Highly significant interactions were selected using the p-value from the likelihood ratio test corrected for the number of individual loci (31 loci) investigated why only interactions with a p-value of < 0.002 were considered significant. As possibly 9 combinations of genotypes exist for each pair of genes we have for the survival part reported hazard ratio estimates for each combination of genotypes with the combination of the most frequent homocygotes as reference. For the gene-expression data where a Bonferroni corrected p-value < 0.05 was considered significant.

## Results

### Study population

The patient population consisted of 139 FL and 216 DLBCL patients with a median age of 63 years including 53% males. The median OS was 75.5 months (58.7 months for DLBCL and 101.7 months for FL). In this population, age (p < 0.0001) and IPI (p < 0.0001), but not sex (p = 0.39) were prognostic markers for OS. The control group consisted of 307 healthy blood donors. Of the controls 46% were males and the median age was 41 years.

### Genotypes and their influence on B-NHL risk

For the *IL6* (rs1800796) SNPs we observed a marginally significant deviation from Hardy-Weinberg equilibrium in the control group (p = 0.042, not corrected for multiple testing). Retesting did not suggest technical problems why this SNP was included in analysis. For the remaining 49 SNPs, no deviations from Hardy-Weinberg equilibrium were observed in the control group. Linkage analysis of the *TNFA*, *IL1B* and *IL10* loci as expected revealed a high degree of linkage disequilibrium (LD) (see S1 Fig).

Table 1 summarizes the significant findings in the univariate analysis. Seven SNPs were solely associated with DLBCL; *TAP2* (rs241447) odds ratio (OR)_DLBCL_ = 0.61 (0.44–0.84), *TLR9* (rs5743836) OR_DLBCL_ = 0.66 (0.44–0.99), *IL4* (rs2243248) OR_DLBCL_ = 1.62 (1.00–2.63), *IL2RA* (rs2104286) OR_DLBCL_ = 0.74 (0.55–0.98), *FCGR2A* (rs1801274) OR_DLBCL_ = 1.37(1.07–1.77), *TNFA* (rs1800629) OR_DLBCL_ = 1.46 (1.06–2.00), *IL10RB* (rs1058867) OR_DLBCL_ = 0.68 (0.53–0.89). Four SNPs were associated solely with FL; *GALNT12* (rs10987898) OR_FL_ = 0.70 (0.49–1.00), *IL12A* (rs485497) OR_FL_ = 0.71 (0.53–0.96), *IL1B* (rs1143627) OR_FL_ = 0.65 (0.48–0.89), *IL1B* (rs16944) OR_FL_ = 0.67 (0.49–0.92). Two SNPs in the *TNFA* locus were associated with both DLBCL and FL, the *TNFA* (rs1799724) and the *TNFA* (rs1799964). OR for all included SNPs are reported in S2, S3 and S4 Tables. Haplotype analysis did not add significant strength to the single gene analysis (data not shown).

**Table 1:** SNPs significantly associated with disease risk in univariate analysis.

			DLBCL+FL			DLBCL			FL		
SNP	Allele	Controls	Patients	OR (95% CI)	p-value	Patients	OR (95% CI)	p-value	Patients	OR (95% CI)	p-value
*TAP2* (rs241447)	A	440	479	1.00	**0.020**	298	1.00	**0.003**	181	1.00	0.663
	G	160	127	0.73 (0.56–0.95)		66	0.61 (0.44–0.84)		61	0.93 (0.66–1.30)	
*TLR9* (rs5743836)	T	523	593	1.00	**0.025**	354	1.00	**0.045**	239	1.00	0.121
	C	85	65	0.67 (0.48–0.95)		38	0.66 (0.44–0.99)		27	0.70 (0.44–1.10)	
*IL4 (rs2243248)*	T	575	617	1.00	0.178	364	1.00	**0.049**	253	1.00	0.934
	G	35	51	1.36 (0.87–2.12)		36	1.62 (1.00–2.63)		15	0.97 (0.52–1.82)	
*IL2RA* (rs2104286)	A	427	508	1.00	0.059	310	1.00	**0.037**	198	1.00	0.399
	G	183	172	0.79 (0.62–1.01)		98	0.74 (0.55–0.98)		74	0.87 (0.63–1.20)	
*TNFA* (rs1799724)	C	559	614	1.00	**<0.001**	370	1.00	**<0.001**	244	1.00	**0.001**
	T	51	10	0.18 (0.09–0.36)		6	0.18 (0.08–0.42)		4	0.18 (0.06–0.50)	
*FCGR2A* (rs1801274)	T	310	326	1.00	0.504	170	1.00	**0.014**	156	1.00	0.057
	C	300	340	1.08 (0.87–1.34)		226	1.37 (1.07–1.77)		114	0.76 (0.57–1.01)	
*TNFA* (rs1800629)	G	509	539	1.00	**0.044**	318	1.00	**0.019**	221	1.00	0.426
	A	101	143	1.34 (1.01–1.77)		92	1.46 (1.06–2.00)		51	1.16 (0.80–1.69)	
*TNFA (rs1799964)*	T	474	490	1.00	**<0.001**	301	1.00	**<0.001**	189	1.00	**0.003**
	C	134	68	0.49 (0.36–0.67)		41	0.48 (0.33–0.70)		27	0.51 (0.32–0.79)	
*GALNT12* (rs10987898)	T	424	465	1.00	0.218	273	1.00	0.776	192	1.00	**0.047**
	G	170	159	0.85 (0.66–1.10)		105	0.96 (0.72–1.28)		54	0.70 (0.49–1.00)	
*IL10RB* (rs1058867)	A	338	421	1.00	**0.004**	256	1.00	**0.005**	165	1.00	0.073
	G	266	237	0.72 (0.57–0.90)		138	0.68 (0.53–0.89)		99	0.76 (0.57–1.03)	
*IL12A* (rs485497)	G	313	370	1.00	0.053	216	1.00	0.277	154	1.00	**0.024**
	A	297	282	0.80 (0.64–1.00)		178	0.87 (0.67–1.12)		104	0.71 (0.53–0.96)	
*IL1B* (rs1143627)	T	377	466	1.00	**0.007**	273	1.00	0.071	193	1.00	**0.007**
	C	231	208	0.73 (0.58–0.92)		131	0.78 (0.60–1.02)		77	0.65 (0.48–0.89)	
*IL1B (rs16944)*	G	374	464	1.00	**0.014**	273	1.00	0.105	191	1.00	**0.012**
	A	230	214	0.75 (0.60–0.94)		135	0.80 (0.62–1.05)		79	0.67 (0.49–0.92)	

For DLBCL, we observed significant interaction between combinations of *IL10* (rs1800796) * *IL4RA* (rs1801275) (p = 0.0002), *IL10* (rs1800896) * *IL1RN* (rs2637988) (p = 0.001), and *TNFA* (rs1799964) * *TLR9* (rs5743836) (p = 0.0018) and for FL; *IL4RA* (rs1805011) * *IL4* (rs2243248) (p = 0.0002), *IL1B (rs1143627)* * *MBL2* (rs12780112) (p = 0.0014) and the *CTLA4* (rs231775) * *IL4RA* (rs1805010) (p = 0.0018).

### Survival analysis

**Fig 1:**
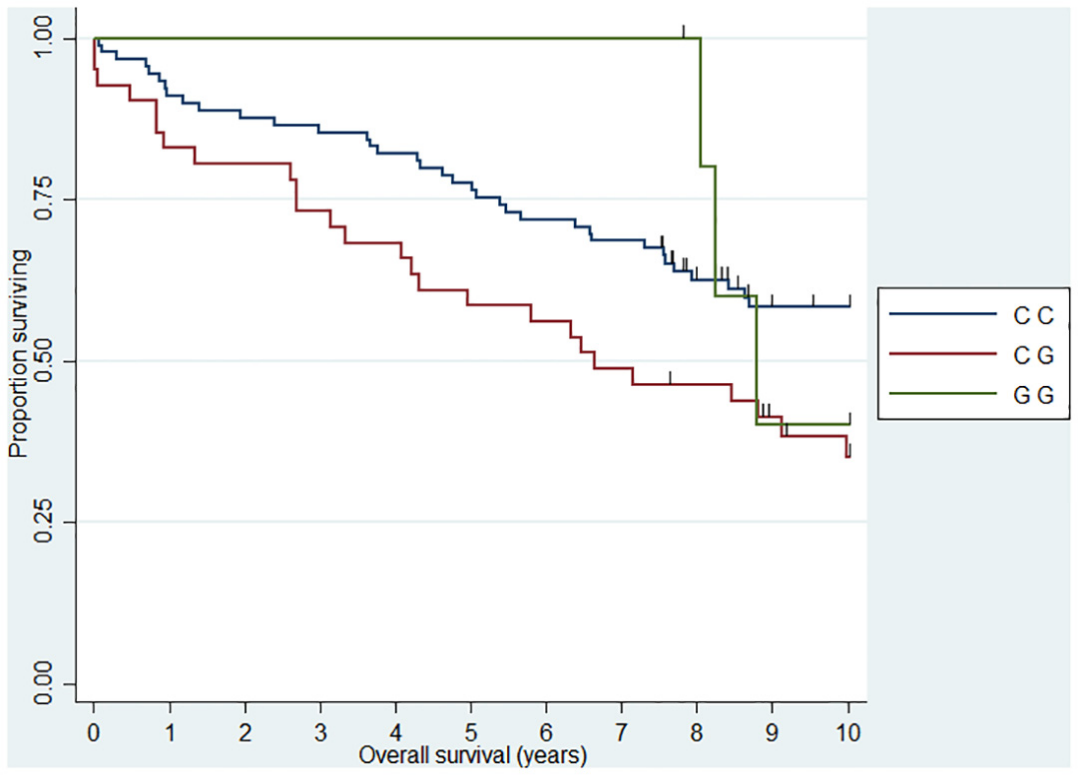
Association between FL 10 year overall survival and *CHI3L1* (rs4950928). HR_CG_ = 2.04 (1.17–3.54)

**Fig 2:**
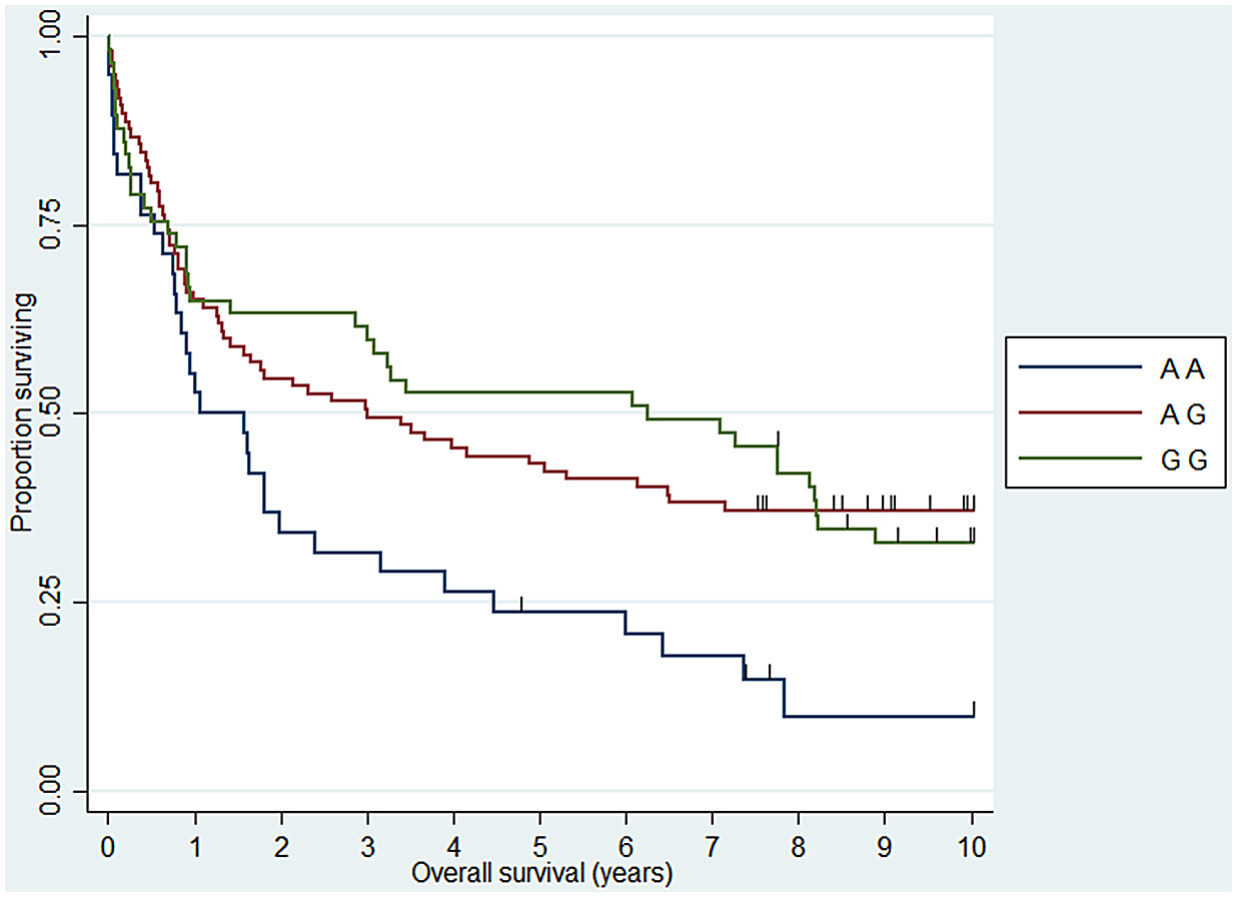
Association between DLBCL 10 year overall survival and *IL12A* (rs485497). HR_AA_ = 1.76 (1.06–2.90).

**Fig 3:**
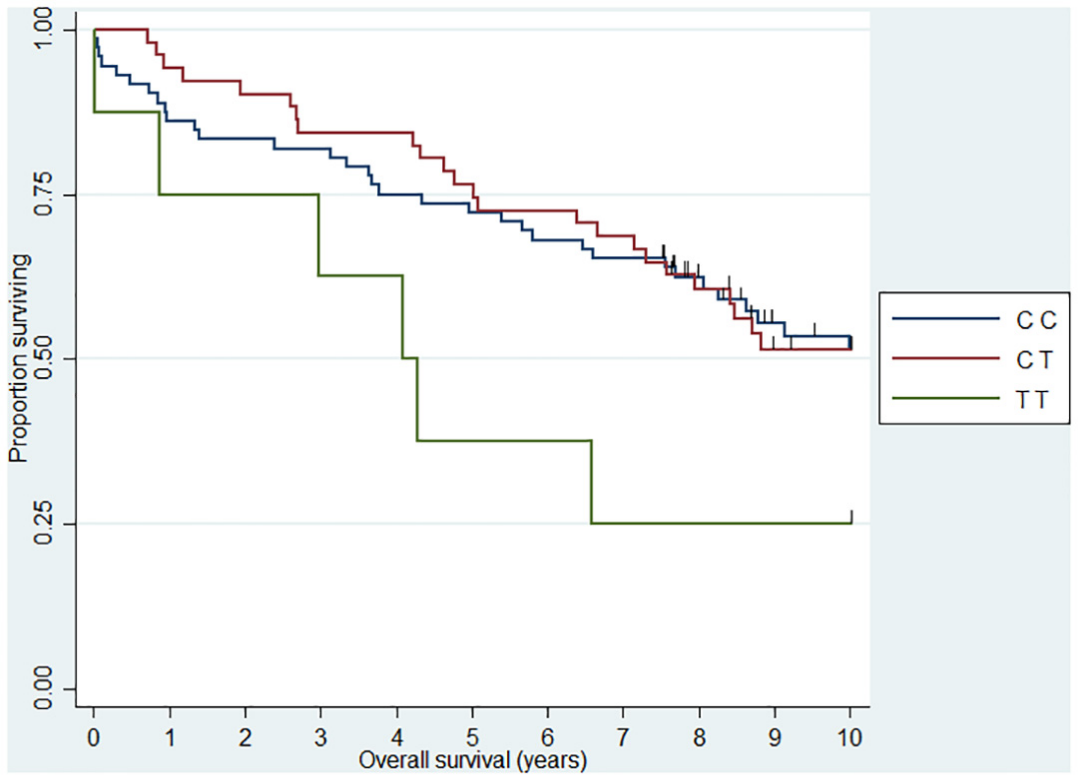
Association between FL 10 year overall survival and *CX3CR1* (rs373379). HR_TT_ = 4.21 (1.67–10.61).

**Fig 4:**
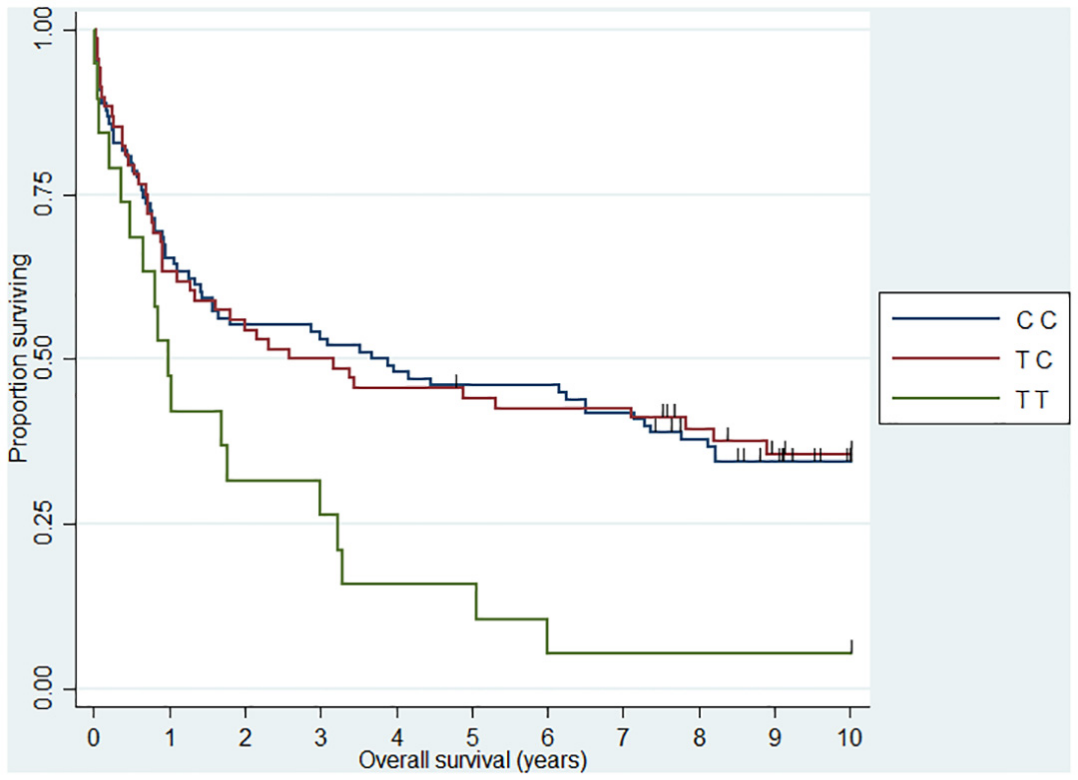
Association between DLBCL 10 year overall survival and *IL5* (rs2069812). HR_TT_ = 1.94 (1.12–3.39).

**Fig 5:**
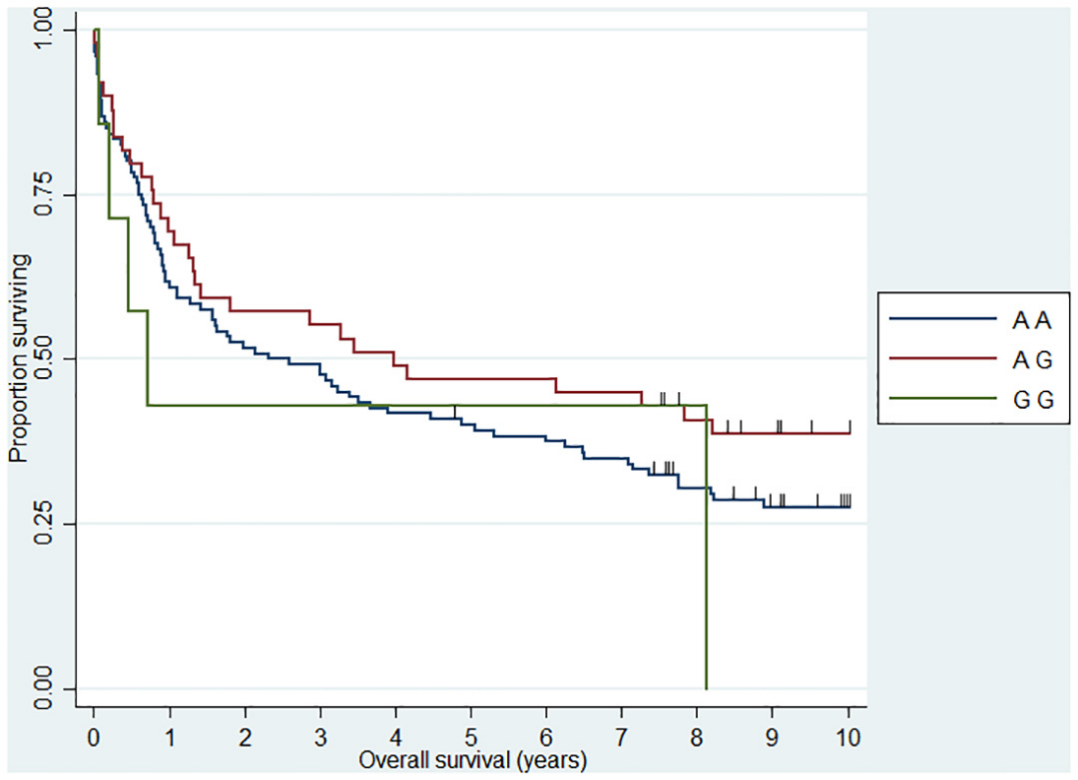
Association between DLBCL 10 year overall survival and *TAP2* (rs241447). HR_GG_ = 3.17 (1.21–8.32).

**Fig 6:**
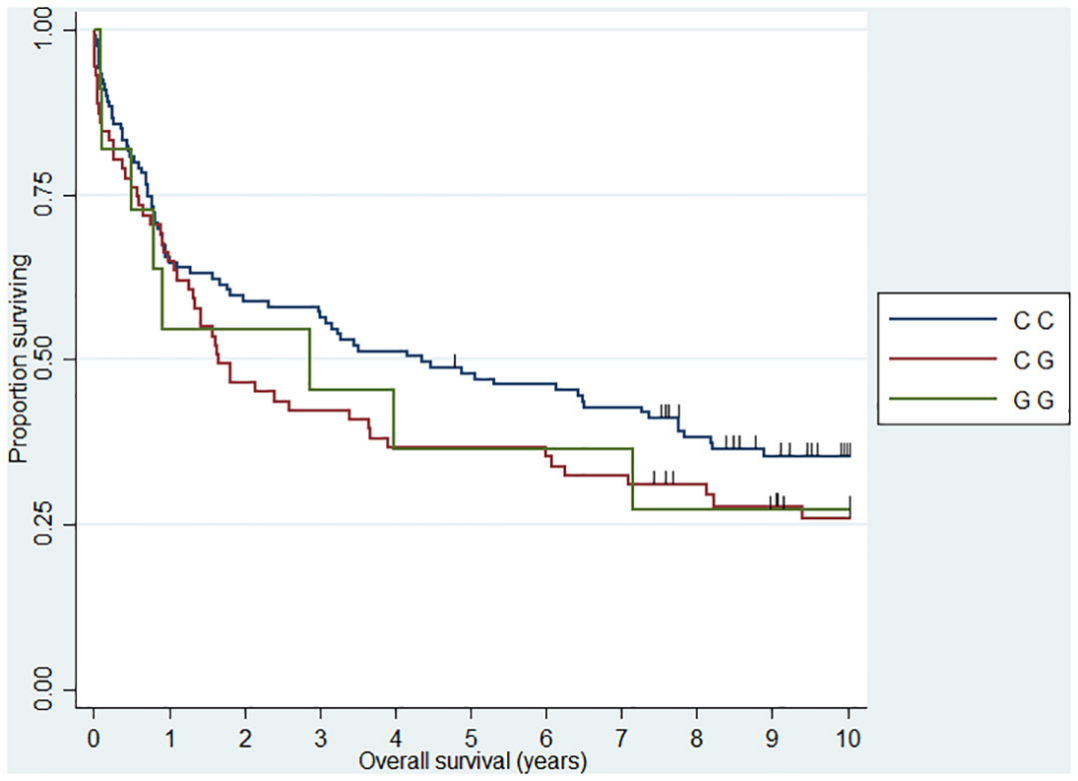
Association between DLBCL 10 year overall survival and MBL2 (rs7096206). HR_CG_ = 1.47 (1.02–2.13)

Univariate 10 year OS analysis of all cases revealed significant association with five SNPs (see Table 2). For DLBCL five SNPs were significantly related to OS; *TAP2* (rs241447) hazard ratio (HR)_GG_ = 3.17 (1.21–8.32), *MBL2* (rs7096206) HR_CG_ = 1.47 (1.02–2.13), *IL5* (rs2069812) HR_TT_ = 1.94 (1.12–3.39), *CX3CR1* (rs373379) HR_TT_ = 2.01 (1.13–3.57), and *IL12A* (rs485497) HR_AA_ = 1.76 (1.06–2.90) (see Table 3). For the FL subgroup two SNPs was associated with survival; *CHI3L1* (rs4950928) HR_CG_ = 2.04 (1.17–3.54) and *CX3CR1* (rs3732379) HR_TT_ = 4.21 (1.67–10.61) (See Table 4). Figs 1–6 provides Kaplan-Meier estimates of the most notable associations. *TNFA* haplotype analysis revealed a deleterious effect of the (rs1799964, rs1799724, rs1800629) CCG haplotype for combined FL and DLBCL and DLBCL alone but not for the FL group alone (HR_B-NHL_ = 1.62 (1.14–2.31), HR_DLBCL_ = 1.74 (1.14–2.66)). HR for all included SNPs are reported in S5, S6 and S7 Tables.

**Table 2:** SNPs associated with 10 year overall survival for B-NHL. The model is adjusted for sex, IPI and treatment.

SNP	Genotype	n	HR (95% CI)	p-value
*SELE* (rs5361)	A A	247	1.00	
	C A	57	0.87 (0.59–1.28)	0.475
	C C	10	0.20 (0.05–0.81)	**0.024**
*IL1RN* (rs419598)	T T	146	1.00	
	C T	90	1.45 (1.03–2.04)	**0.034**
	C C	18	1.16 (0.60–2.27)	0.658
*CX3CR1* (rs3732379)	C C	173	1.00	
	C T	126	0.91 (0.66–1.24)	0.542
	T T	25	2.01 (1.25–3.24)	**0.004**
*TNFA* (rs1799964)	T T	207	1.00	
	C T	60	1.51 (1.02–2.23)	**0.039**
	C C	1	10.67 (1.27–89.72)	**0.029**
*IL12A* (rs485497)	G G	99	1.00	
	A G	156	1.27 (0.90–1.79)	0.169
	A A	59	1.79 (1.18–2.70)	**0.006**

**Table 3:** SNPs associated with 10 year overall survival for DLBCL. The model is adjusted for sex, IPI and treatment.

SNP	Genotype	n	HR (95% CI)	p-value
*TAP2* (rs241447)	A A	117	1.00	
	A G	49	0.88 (0.56–1.38)	0.572
	G G	7	3.17 (1.21–8.32)	**0.019**
*MBL2* (rs7096206)	C C	118	1.00	
	C G	70	1.47 (1.02–2.13)	**0.041**
	G G	10	1.15 (0.52–2.57)	0.727
*IL5* (rs2069812)	C C	98	1.00	
	T C	66	1.06 (0.70–1.61)	0.778
	T T	18	1.94 (1.12–3.39)	**0.019**
*CX3CR1* (rs3732379)	C C	102	1.00	
	C T	76	0.86 (0.59–1.27)	0.456
	T T	17	2.01 (1.13–3.57)	**0.018**
*IL12A* (rs485497)	G G	54	1.00	
	A G	95	1.04 (0.67–1.60)	0.874
	A A	39	1.76 (1.06–2.90)	**0.028**

**Table 4:** SNPs associated with 10 year overall survival for FL. The model is adjusted for sex, IPI and treatment.

SNP	Genotype	n	HR (95% CI)	p-value
*CHI3L1* (rs4950928)	C C	89	1.00	
	C G	39	2.04 (1.17–3.54)	**0.012**
	G G	6	0.33 (0.09–1.27)	0.106
*CX3CR1* (rs3732379)	C C	71	1.00	
	C T	50	0.90 (0.51–1.59)	0.723
	T T	8	4.21 (1.67–10.61)	**0.002**

Gene-gene interactions analysis revealed several gene combinations significantly related to adjusted OS. For DLBCL; *IL4RA* (rs1805010) * *IL10* (rs1800890) (p = 0.00021), *FCGR2A* (rs1801274) * *IL1RN* (rs419598) (p = 0.0003), *IL10* (rs1800896) * *IL4RA* (rs1805010) (p = 0.0004), *IL10* (rs1800896) * *IL1RA* (rs419598) (p = 0.0006), *TNFRSF1B* (rs1061622) * *IL10* (rs1800890) (p = 0.0008), *TNFRSF1B* (rs1061622) * MBL2 (rs7096206) (p = 0.0009), *CX3CR1* (rs373379) * *IL12RB1* (rs2305742) (p = 0.0011), *IL10* (rs1800872) * *IL2* (rs2069762) (p = 0.001) and *IL10* (rs1800871) * *IL4RA* (rs1805010) (p = 0.001). Analysis of the FL subgroup revealed interactions for the *IL1RN* (rs2637988) * *IL4* (rs2243248) (p = 0.0004), *TNFRSF1B* (rs1061622) * IL2RA (rs2104286) (p = 0.0006)). HR for specific genotype combinations is reported in S8, S9 and S10 Tables.

### Gene expression analysis in normal B-cell subsets and DLBCL lymphoma cells

**Fig 7:**
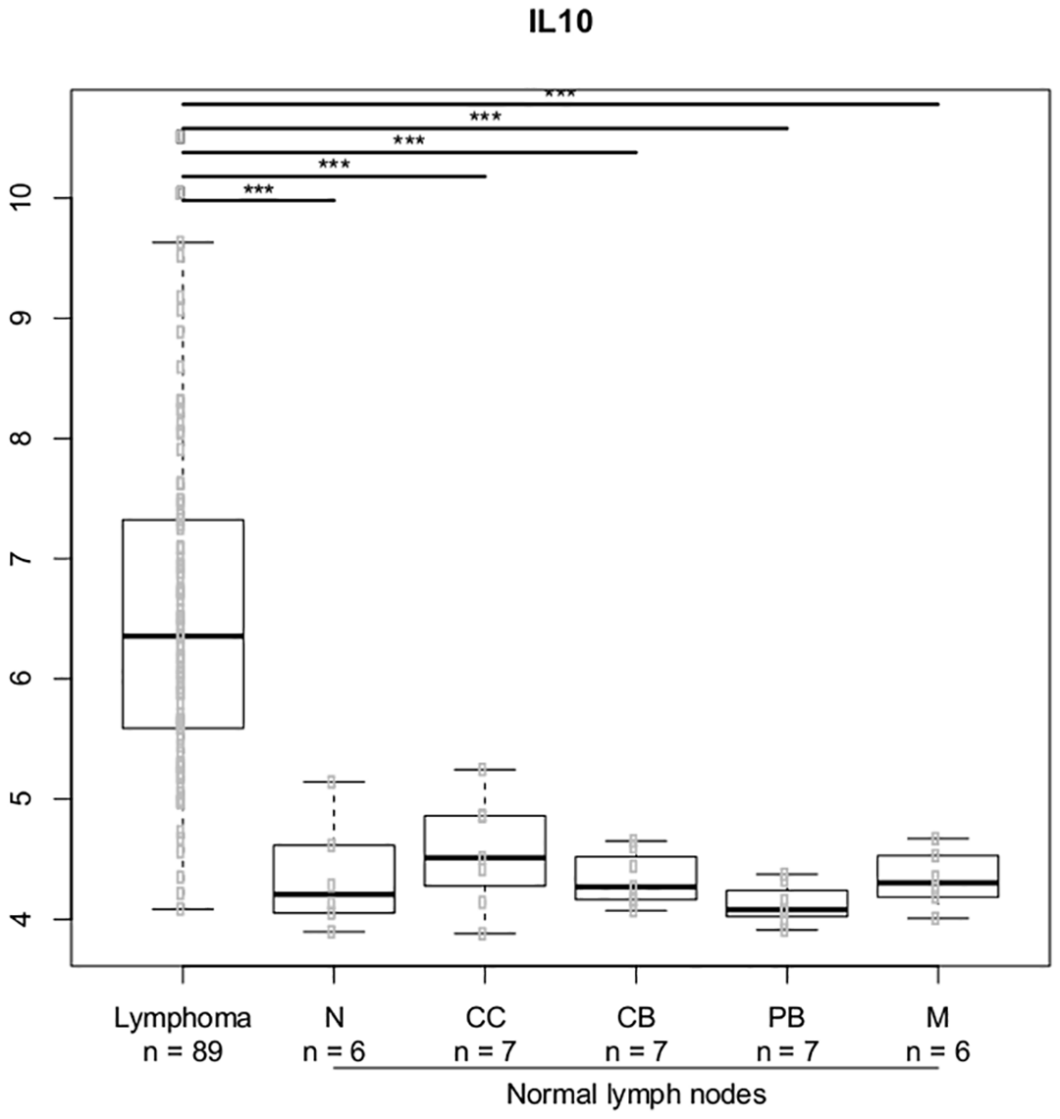
Gene expression for the *IL10* gene. The asterisk represents significant difference in expression after Bonferroni correction (*** = p < 0.001, ** = p< 0.01, * = p < 0.05). Gene expression is presented on a log2 scale. Lymphoma: DLBCL B-cells, N: Naïve B-cells, CC: Centrocytes, CB: Centroblasts, PB: Plasmablasts, M: Memory B-cells.

**Fig 8:**
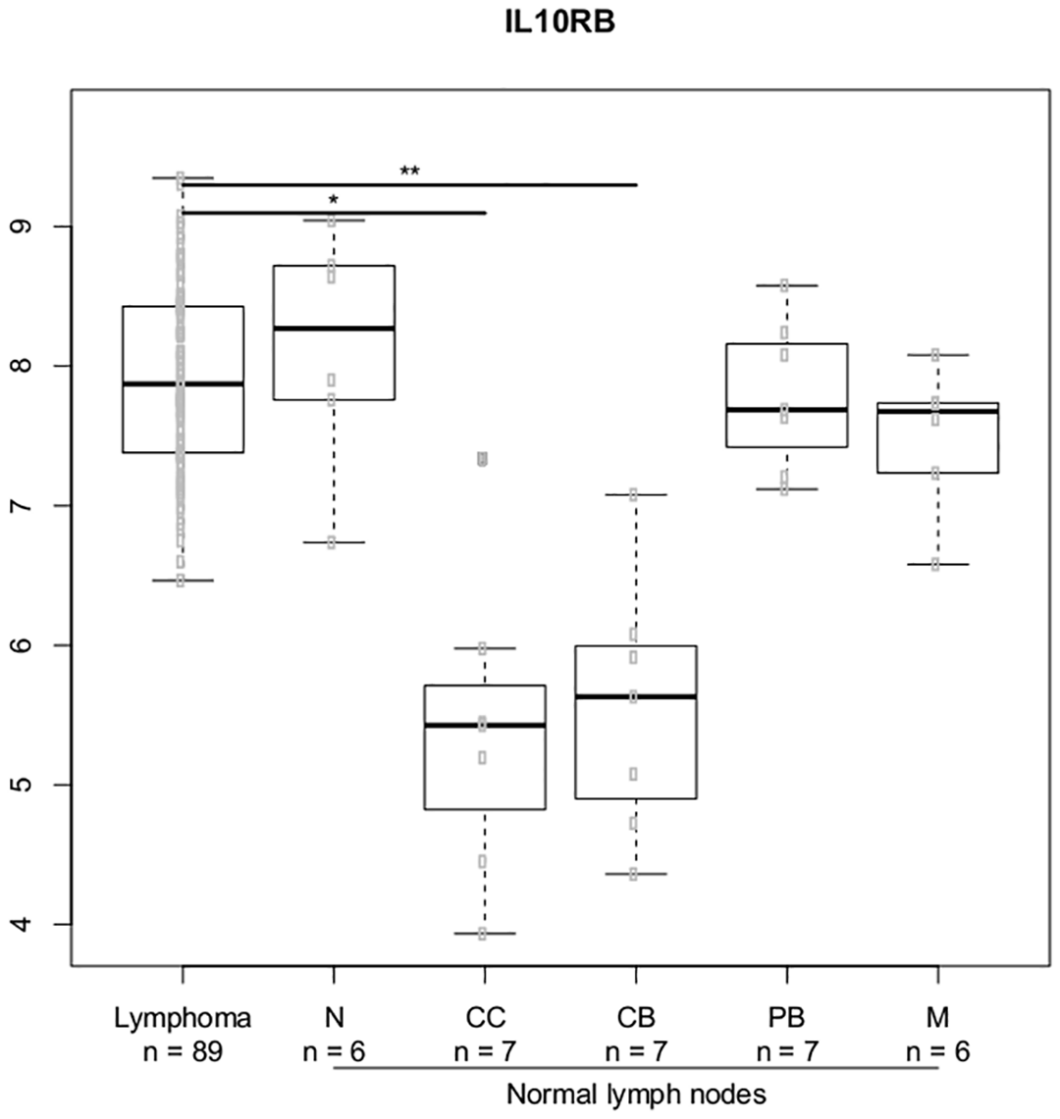
Gene expression for the *IL10RB* gene. , The asterisk represents significant difference in expression after Bonferroni correction (*** = p < 0.001, ** = p< 0.01, * = p < 0.05). Gene expression is presented on a log2 scale. Lymphoma: DLBCL B-cells, N: Naïve B-cells, CC: Centrocytes, CB: Centroblasts, PB: Plasmablasts, M: Memory B-cells.

**Fig 9:**
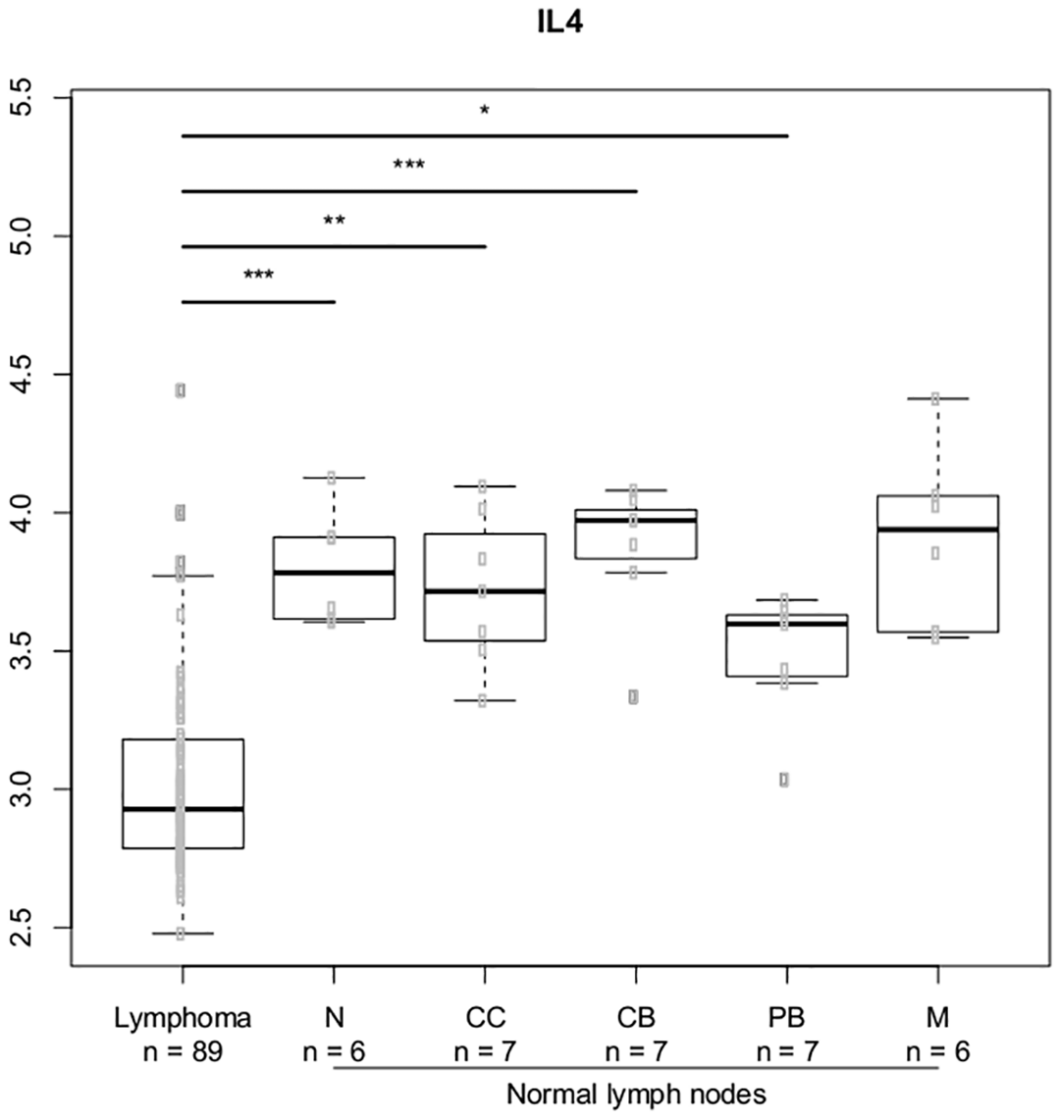
Gene expression for the *IL4* gene. The asterisk represents significant difference in expression after Bonferroni correction (*** = p < 0.001, ** = p< 0.01, * = p < 0.05). Gene expression is presented on a log2 scale. Lymphoma: DLBCL B-cells, N: Naïve B-cells, CC: Centrocytes, CB: Centroblasts, PB: Plasmablasts, M: Memory B-cells.

**Fig 10:**
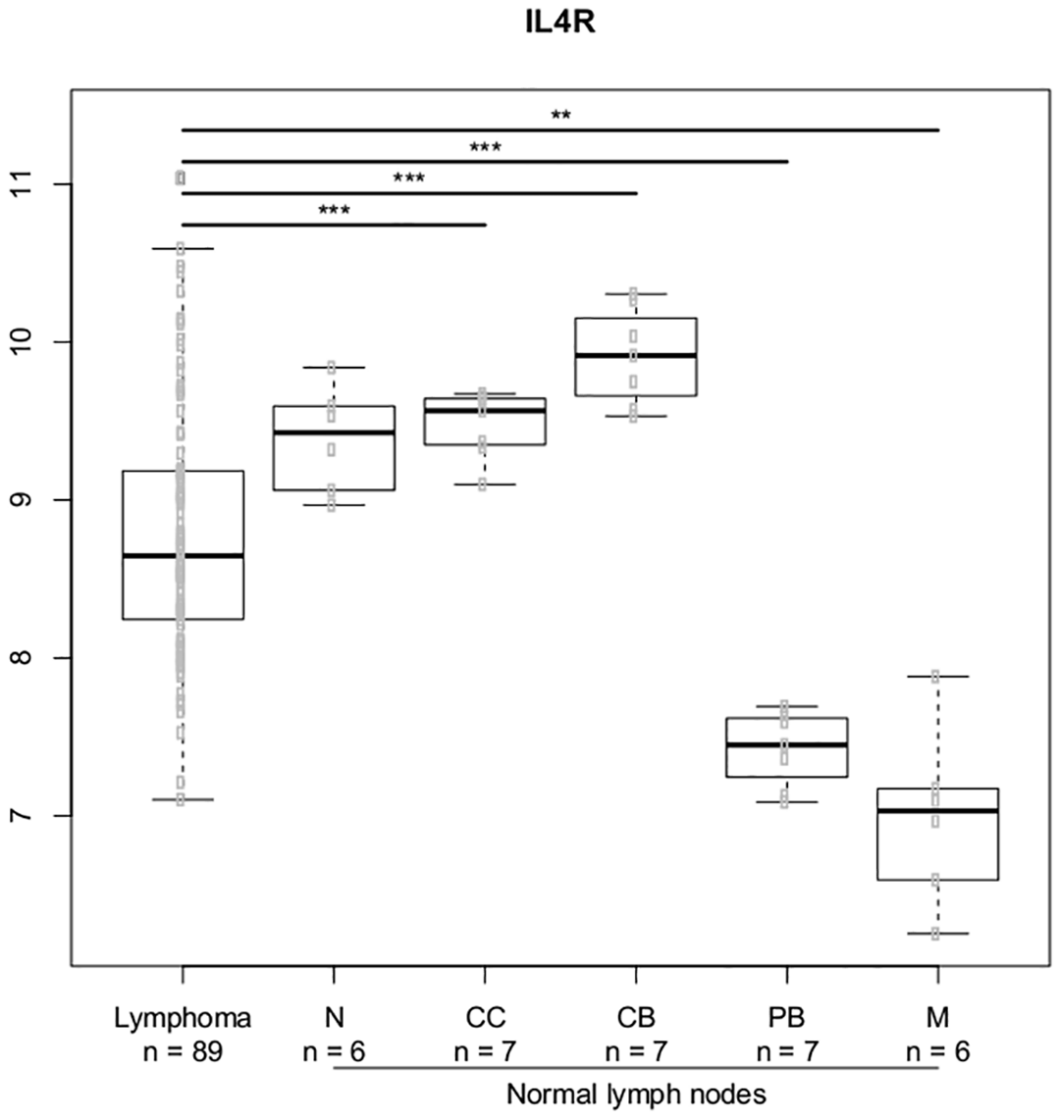
Gene expression for the *IL4R* gene. The asterisk represents significant difference in expression after Bonferroni correction (*** = p < 0.001, ** = p< 0.01, * = p < 0.05). Gene expression is presented on a log2 scale. Lymphoma: DLBCL B-cells, N: Naïve B-cells, CC: Centrocytes, CB: Centroblasts, PB: Plasmablasts, M: Memory B-cells.

We selected the most significant genes from the gene-gene interaction analysis and analyzed the expression of these genes in the normal lymph node B-cell hierarchy and DLBCL lymphoma cells. We explored genes related to the IL-4 and IL-10 cytokines; Figs 7–10 illustrates differences in expression between normal germinal centre (GC) B-cells (defined as centrocytes (CC) and centroblasts (CB)) and non-GC subtypes (naïve (N), plasmablasts (PB), memory (M) B-cells) and DLBCL cells using Bonferroni corrected p-values. The *IL10* gene (Fig 7) and the *IL10RB* (Fig 8) gene were up regulated in DLBCL cells when compared to normal GC subtypes. The increased expression of *IL10* was only seen in DLBCL cells whereas increased *IL10RB* expression could also be shown in pre- and post germinal center subpopulations. The *IL4* gene was equally expressed in all normal B-cell subpopulations (Fig 9) whereas the expression in DLBCL cells was slightly down regulated, a similar expression profile was seen for the *IL4R* gene (Fig 10) however this gene was also found to be down regulated in post GC subpopulations.

## Discussion

DLBCL and FL share a common dependence of the inflammatory tumor microenvironment for their proliferation and growth [7] however we generally observed a specific association with disease risk supporting DLBCL and FL being two different diseases with respect to genetic risk factors.

Interleukins (IL) and toll-like receptors (TLR) play a major role in B-cell differentiation and proliferation. The *TNFA* gene have been intensively studied and in accordance with others, including the large pooled Interlymph study and a recent GWAS study including largely the same patients [10,37] we found the *TNFA* (rs1800629) A allele associated with increased risk of DLBCL (OR = 1.46 (1.06–2.00)). The functional *IL1B* (rs1143627) C allele and *IL1B* (rs16944) A allele were associated with a decreased risk of FL but not DLBCL, a trend for an association with the *IL1B* gene was observed in the Interlymph study [10], however substantial difference was observed between the included cohorts.[10] The *IL10RB* (rs1058867) SNP was earlier investigated in a pooled study [12] reporting an association with FL; results we failed to confirm, whereas we observed a significant association in DLBCL where a decreased risk was shown for DLBCL carries of the G allele. Inappropriate TLR9 activation has been reported in malignant B-cells [38] and we observed a decreased risk of B-NHL (OR = 0.67) and DLBCL (OR = 0.66) for carriers of the *TLR9* (rs5743836) C allele. This was in agreement with observations by Carvalho et al. [39] and suggests a role for TLR signalling in lymphomagenesis.

Among other notable findings, the (rs241447) SNP in the *TAP2* gene, coding for a HLA-II associated transport protein [40], was significantly associated with decreased risk of DLBCL, OR = 0.61 (0.44–0.84). Vijai et al. reported an adjacent SNP in the *TAP2* gene to be associated with risk of DLBCL and FL, a finding confirmed by Cerhan et al. [23,40]. The cumulative findings emphasize the possible importance of genetic variation in the HLA-II region and further evaluation of the *TAP2* gene in larger cohorts is awaited. The *GALNT12* gene is linked to pathological aberrant glycosylation, and was associated with outcome in FL in a recent GWAS [41] study including 244 FL cases. We observed a decreased risk of FL for carries of the *GALNT12* (rs10987898) G allele whereas there was no apparent effect on DLBCL risk, supporting a specific role for this gene in the pathogenesis of FL and emphasising the need to explore this gene in relation to the glycosylation patterns in FL B-cells. Another intensively studied locus [13,15,42] is the *FCGR2A* gene. The *FCGR2A* gene encodes a low affinity Immunoglobulin-G (IgG) receptor (FcγRIIa) and genetic variation affects inflammatory cytokine production [43]. The association between the *FCGR2A* (rs1801274) SNP and DLBCL was also confirmed in our population (OR = 1.37 (1.07–1.77)) whereas the association with FL was not statistically significant (OR = 0.76 (0.57–1.01)).

When we analyzed 10 year OS, we observed an association between the *CHI3L1* (rs4950928) C allele and a worse outcome in FL (CG genotype compared to the CC genotype)—the rare GG genotype group was unfortunately small and did not add sufficient information as presented the Kaplan-Meier plot (Fig 1). The *CHI3L1* gene encodes the pro-inflammatory YKL-40 protein [44] and we have previously reported circulating levels of YKL-40 to be associated with dismal outcome in NHL [45] and the existence of the functional (rs4950928) SNP in the *CHI3L1* gene affecting serum-YKL-40 [33], however to the best of our knowledge the *CHI3L1* gene have not previously been investigated in this setting. The *SELE* gene encodes the selectin-E molecule expressed on endothelial cells known to promote metastasis [46]; the *SELE* (rs5361) SNP was found to be associated with OS in FL and DLBCL in a recent study [47] and we found an equal effect on the combined DLBCL and FL cohort, however, the effect was not obvious when analyzed at specific diagnostic entity levels. *IL12A* (rs485497) (Fig 2) was associated with decreased risk in FL, as well as outcome in DLBCL and we also confirmed the earlier suggested association between B-NHL outcome and the *CX3CR1* gene [47] (Fig 3) and we did observe a novel association between the *IL5* (rs2069812), encoding B-cell growth factor IL-5 [48], and outcome of DLBCL ((HR = 1.94 (1.12–3.39)) for carriers of the TT genotype, Fig 4). Surprisingly, we observed a DLBCL specific association between the *TAP2* (rs241447) gene in GG genotype and decreased OS however the G allele is significantly associated with decreased risk of DLBCL (OR = 0.61 (0.44–0.84)) we do not have an explanation for this findings, but it could suggest that this protein could play a different role in disease initiation and response to treatment (Fig 5). The suggested effect are however in line with the general believe of genes in the HLA-II region of chromosome 6p as one of the most interesting areas in the genome in relation to B-cell lymphomas and our findings of a possible relation to OS in DLBCL must encourage to further studies of this region. The *MBL2* gene has been studied in a number of malignant and infectious diseases with conflicting results. The MBL2 (rs7096206) is associated with risk of hepatocellular carcinoma [49] and febrile neutropenia [50] however the genotype have not been studied in relation to NHL [51]. Whether the effect of this genotype on DLBCL survival (Fig 6) is related to disease progression or increased risk of infectious complication needs to be investigated in future studies.

Growing evidence suggest, that inflammatory mediators act in networks why co-occurrence of genotypes -gene-gene interaction -analysis needs to be considered in complex diseases as B-NHL [24,52]. Of most notable interest, was a highly significant interaction between two risk alleles; *IL10* (rs1800872) and *IL4RA* (rs1801275). IL-4 and IL-10 play a major role in the GC reaction, B-cell proliferation is enhanced by IL-10 in the presence of IL-4 and both cytokines are involved in the class switch recombination process [53–55]. We further explored gene-gene interactions in relation to FL and DLBCL specific OS. Most notably, we discovered a highly significant (p = 0.0002) interaction between *IL10* (rs1800890) and *IL4RA* (rs1805010) in DLBCL. Carriers of *IL10* (rs1800890) *AA* in combination with the *IL4RA* (rs1805010) *AA* genotype had a significantly improved outcome (HR = 0.11 (0.20–0.50)). Several SNPs in the *IL4*, *IL4R* and *IL10* genes has been proposed associated with outcome in earlier studies [20]. Analysis of the effect of these individual genes have been somewhat contradictory however the results of attempts to include these loci in multi-gene models [47], including ours, argues for a combined effect of these loci not readily identified in a single gene model emphasizing the interaction between these cytokines to be of importance and in need of exploration in future studies. For FL the most notable effect of gene-gene interactions was seen for interaction between the *IL1RN* (rs2637988) and *IL4* (rs2243248) (p = 0.0004). As it was the case for DLBCL, a SNP related to the *IL-4* cytokine was involved in gene-gene interactions in the FL group. We did explore survival estimates for gene-gene combinations (S8, S9 and S10 Tables), however risk estimates are imprecise since the number of events in each group was small.

In order to study the functional aspect of these genes in the B-cell hierarchy, we combined the SNP study with analysis of gene expression in normal B-cell subpopulations and DLBCL lymphoma cells. We restricted the analysis to the most notable genes from the gene-gene expression analysis which suggested a prominent effect of IL-4 and IL-10 related genes. Increased expression of IL-10 related genes has earlier been shown in chronic lymphocytic leukaemia (CLL) and in Waldenström's macroglobulinemia [56]. We found a up regulation of the *IL10* and the *IL10RB* gene in DLBCL cells when compared to normal GC B-cell subpopulations supporting the hypothesis that this cytokine/receptor could play a role for the survival of germinal centre derived lymphomas, as supported by the earlier findings in CLL [56] and further supporting the protective effect of the low producer *IL10* (rs1800872) AA genotype as well as the protective effect of the *IL10RB* (rs1058867) G allele [10,12]. The *IL4* gene was slightly down regulated in DLBCL cells when compared to all normal B-cell subpopulations whereas the *IL4R* gene was down regulated in post GC B-cell subpopulations suggesting that these genes could be important at different stages of the stepwise oncogenetic process [57] and further suggesting that such genes with oncogenetic potential may be important in the multi step process of malignant transformation even if they are not found to be expressed in malignant tissue [9]. These findings needs to explored further by investigating the expression of these genes in the different B-cell subpopulation thought to be the cell of origin of the investigated B-cell malignancy [31,58]. The functional properties of these pathways obviously needs further investigation; as an example, the functional properties of *IL4* gene and the distal *IL10* promotor variations still remains largely unknown and so does the combined effect of these interactions on protein level [59]. Our study holds some limitations providing a possible bias. We do acknowledge that the sample size in our study was too small for in depth investigation of gene-gene interactions making our risk estimates less precise. We however do encourage that the findings from our exploratory investigation of gene-gene interactions is investigated in a larger cohort, preferably a multicenter study. The age distribution in the control group and patient group was not identical and the sample material differed between groups. We found no association between age and genotypes within the groups and we included a cohort with no reported BM involvement, however these differences between groups still could have introduced bias to our results.

## Conclusion

We analysed 50 SNPs in inflammatory response genes with respect to risk and outcome in B-NHL. We reported a significant association between thirteen SNPs and risk and reported seven SNPs associated with outcome. Moreover we here suggest a gene-gene interaction effect in relation to risk and survival and when we combined these findings with gene expression analysis in normal and malignant B-cells we suggest that these inherited variations could play a role at different stages of the multi step process of B-cell oncogenesis. These findings further strengthened the discovered association between interacting key inflammatory response genes and B-lymphoma and we propose further studies exploring the functional aspects of these interactions. As for earlier studies on genetics in lymphoma, our results were not always consistent with findings in other B-NHL cohorts. This can be a result of sample size as well as ethnical differences between study populations [51] although we have a well defined, ethnically homogenous population with a long follow up period. Despite limitations of our study, we believe that our findings confirms some of the earlier reported findings and adds new knowledge to the immunogenetics as an important factor in relation to risk and outcome in B-NHL.

## Supporting Information

S1 Fig LD plots for the TNFA, IL-1 and IL-10 loci. (TIF)

S1 Table Assay information 50SNPs genotyped in 31 genes. (DOCX)

S2 Table Association between SNPs and B-NHL risk (DOCX)

S3 Table Association between SNPs and DLBCL risk. (DOCX)

S4 Table Association between SNPs and risk of FL (DOCX)

S5 Table Assoication between SNPs and 10 year overall survival for B-NHL. The model is adjusted for sex, IPI and treatment. (DOCX)

S6 Table Assoication between SNPs and 10 year overall survival for DLBCL. The model is adjusted for sex, IPI and treatment. (DOCX)

S7 Table Assoication between SNPs and 10 year overall survival for FL. The model is adjusted for sex, IPI and treatment. (DOCX)

S8 Table Gene-gene interactions in relation to overall survival in all patients (DOCX)

S9 Table Gene-gene interactions in relation to overall survival in DLBCL (DOCX)

S10 Table Gene-gene interactions in relation to overall survival in FL (DOCX)

S1 Text Supplementary methods. (DOCX)

## References

[1] Shaffer AL, Rosenwald A, Staudt LM. Lymphoid malignancies: the dark side of B-cell differentiation. Nat Rev Immunol. 2002;2: 920–32. 10.1038/nri953 12461565

[2] Küppers R. Mechanisms of B-cell lymphoma pathogenesis. Nat Rev Cancer. 2005;5: 251–62. 10.1038/nrc1589 15803153

[3] Dybkaer K, Bogsted M, Falgreen S, Bodker JS, Kjeldsen MK, Schmitz A, et al Diffuse Large B-Cell Lymphoma Classification System That Associates Normal B-Cell Subset Phenotypes With Prognosis. J Clin Oncol. 2015; 10.1200/JCO.2014.57.7080 PMC4397280 25800755

[4] Jaffe ES. The 2008 WHO classification of lymphomas: implications for clinical practice and translational research. Hematology Am Soc Hematol Educ Program. 2009; 523–31. 10.1182/asheducation-2009.1.523 20008237 PMC6324557

[5] Dave SS, Wright G, Tan B, Rosenwald A, Gascoyne RD, Chan WC, et al Prediction of survival in follicular lymphoma based on molecular features of tumor-infiltrating immune cells. N Engl J Med. 2004;351: 2159–69. 10.1056/NEJMoa041869 15548776

[6] Lenz G, Wright G, Dave SS, Xiao W, Powell J, Zhao H, et al Stromal gene signatures in large-B-cell lymphomas. N Engl J Med. 2008;359: 2313–23. 10.1056/NEJMoa0802885 19038878 PMC9103713

[7] Scott DW, Gascoyne RD. The tumour microenvironment in B cell lymphomas. Nat Rev Cancer. Nature Publishing Group; 2014;14: 517–534. 10.1038/nrc3774 25008267

[8] Howell WM, Rose-Zerilli MJ. Cytokine gene polymorphisms, cancer susceptibility, and prognosis. J Nutr. 2007;137: 194S–199S. Available: http://www.ncbi.nlm.nih.gov/pubmed/17640324 17182825 10.1093/jn/137.1.194S

[9] Green MR, Vicente-Dueñas C, Alizadeh A a, Sánchez-García I. Hit-and-run lymphomagenesis by the Bcl6 oncogene. Cell Cycle. 2014;13: 1831–2. 10.4161/cc.29326 24867153 PMC4111743

[10] Rothman N, Skibola CF, Wang SS, Morgan G, Lan Q, Smith MT, et al Genetic variation in TNF and IL10 and risk of non-Hodgkin lymphoma: a report from the InterLymph Consortium. Lancet Oncol. 2006;7: 27–38. 10.1016/S1470-2045(05)70434-4 16389181

[11] Wang SS, Purdue MP, Cerhan JR, Zheng T, Menashe I, Armstrong BK, et al Common gene variants in the tumor necrosis factor (TNF) and TNF receptor superfamilies and NF-kB transcription factors and non-Hodgkin lymphoma risk. PLoS One. 2009;4: e5360 10.1371/journal.pone.0005360 19390683 PMC2669130

[12] Lan Q, Wang SS, Menashe I, Armstrong B, Zhang Y, Hartge P, et al Genetic variation in Th1/Th2 pathway genes and risk of non-Hodgkin lymphoma: a pooled analysis of three population-based case-control studies. Br J Haematol. 2011;153: 341–50. 10.1111/j.1365-2141.2010.08424.x 21418175 PMC3075370

[13] Wang SS, Cerhan JR, Hartge P, Davis S, Cozen W, Severson RK, et al Common genetic variants in proinflammatory and other immunoregulatory genes and risk for non-Hodgkin lymphoma. Cancer Res. 2006;66: 9771–80. 10.1158/0008-5472.CAN-06-0324 17018637

[14] Skibola CF, Curry JD, Nieters A. Genetic susceptibility to lymphoma. Haematologica. 2007;92: 960–9. Available: http://www.pubmedcentral.nih.gov/articlerender.fcgi?artid=2823809&tool=pmcentrez&rendertype=abstract 17606447 10.3324/haematol.11011 PMC2819165

[15] Purdue MP, Lan Q, Kricker A, Grulich AE, Vajdic CM, Turner J, et al Polymorphisms in immune function genes and risk of non-Hodgkin lymphoma: findings from the New South Wales non-Hodgkin Lymphoma Study. Carcinogenesis. 2007;28: 704–12. 10.1093/carcin/bgl200 17056605

[16] Schoof N, von Bonin F, Zeynalova S, Ziepert M, Jung W, Loeffler M, et al Favorable impact of the interleukin–4 receptor allelic variant I75 on the survival of diffuse large B-cell lymphoma patients demonstrated in a large prospective clinical trial. Ann Oncol. 2009;20: 1548–54. 10.1093/annonc/mdp110 19515749

[17] Hohaus S, Giachelia M, Di Febo A, Martini M, Massini G, Vannata B, et al Polymorphism in cytokine genes as prognostic markers in Hodgkin’s lymphoma. Ann Oncol. 2007;18: 1376–81. 10.1093/annonc/mdm132 17496310

[18] Warzocha K, Ribeiro P, Bienvenu J, Roy P, Charlot C, Rigal D, et al Genetic polymorphisms in the tumor necrosis factor locus influence non-Hodgkin’s lymphoma outcome. Blood. 1998;91: 3574–81. Available: http://www.ncbi.nlm.nih.gov/pubmed/9572991 9572991

[19] Lech-Maranda E, Baseggio L, Bienvenu J, Charlot C, Berger F, Rigal D, et al Interleukin-10 gene promoter polymorphisms influence the clinical outcome of diffuse large B-cell lymphoma. Blood. 2004;103: 3529–34. 10.1182/blood-2003-06-1850 14701701

[20] Kube D, Hua T-D, von Bonin F, Schoof N, Zeynalova S, Klöss M, et al Effect of interleukin-10 gene polymorphisms on clinical outcome of patients with aggressive non-Hodgkin’s lymphoma: an exploratory study. Clin Cancer Res. 2008;14: 3777–84. 10.1158/1078-0432.CCR-07-5182 18559596

[21] Cerhan JR, Ansell SM, Fredericksen ZS, Kay NE, Liebow M, Call TG, et al Genetic variation in 1253 immune and inflammation genes and risk of non-Hodgkin lymphoma. Blood. 2007;110: 4455–63. 10.1182/blood-2007-05-088682 17827388 PMC2234796

[22] Smedby KE, Foo JN, Skibola CF, Darabi H, Conde L, Hjalgrim H, et al GWAS of follicular lymphoma reveals allelic heterogeneity at 6p21.32 and suggests shared genetic susceptibility with diffuse large B-cell lymphoma. PLoS Genet. 2011;7: e1001378 10.1371/journal.pgen.1001378 21533074 PMC3080853

[23] Cerhan JR, Fredericksen ZS, Novak AJ. A Two-Stage Evaluation of Genetic Variation in Immune and Inflammation Genes with Risk of Non-Hodgkin Lymphoma Identifies New Susceptibility Locus in 6p21.3 Region. 2012; 10.1158/1055-9965.EPI-12-0696 PMC3467356 22911334

[24] Cordell HJ. Detecting gene-gene interactions that underlie human diseases. Nat Rev Genet. 2009;10: 392–404. 10.1038/nrg2579 19434077 PMC2872761

[25] Butterbach K, Beckmann L, de Sanjosé S, Benavente Y, Becker N, Foretova L, et al Association of JAK-STAT pathway related genes with lymphoma risk: results of a European case-control study (EpiLymph). Br J Haematol. 2011;153: 318–33. 10.1111/j.1365-2141.2011.08632.x 21418178

[26] Forrest MS, Skibola CF, Lightfoot TJ, Bracci PM, Willett EV, Smith MT, et al Polymorphisms in innate immunity genes and risk of non-Hodgkin lymphoma. Br J Haematol. 2006;134: 180–3. 10.1111/j.1365-2141.2006.06141.x 16740140

[27] Nieters A, Beckmann L, Deeg E, Becker N. Gene polymorphisms in Toll-like receptors, interleukin-10, and interleukin-10 receptor alpha and lymphoma risk. Genes Immun. 2006;7: 615–24. 10.1038/sj.gene.6364337 16971956

[28] Lu Y, Abdou AM, Cerhan JR, Morton LM, Severson RK, Davis S, et al Human leukocyte antigen class I and II alleles and overall survival in diffuse large B-cell lymphoma and follicular lymphoma. ScientificWorldJournal. 2011;11: 2062–70. 10.1100/2011/373876 22125456 PMC3217596

[29] Cerhan JR, Wang S, Maurer MJ, Ansell SM, Geyer SM, Cozen W, et al Prognostic significance of host immune gene polymorphisms in follicular lymphoma survival. Blood. 2007;109: 5439–46. 10.1182/blood-2006-11-058040 17327408 PMC1890834

[30] Habermann TM, Wang SS, Maurer MJ, Morton LM, Lynch CF, Ansell SM, et al Host immune gene polymorphisms in combination with clinical and demographic factors predict late survival in diffuse large B-cell lymphoma patients in the pre-rituximab era. Blood. 2008;112: 2694–702. 10.1182/blood-2007-09-111658 18633131 PMC2556607

[31] Johnsen HE, Bergkvist KS, Schmitz A, Kjeldsen MK, Hansen SM, Gaihede M, et al Cell of origin associated classification of B-cell malignancies by gene signatures of the normal B-cell hierarchy. Leuk Lymphoma. 2013; 1–10. 10.3109/10428194.2013.839785 23998255

[32] Rasmussen T, Haaber J, Dahl IM, Knudsen LM, Kerndrup GB, Lodahl M, et al Identification of translocation products but not K-RAS mutations in memory B cells from patients with multiple myeloma. Haematologica. 2010;95: 1730–7. 10.3324/haematol.2010.024778 20511669 PMC2948099

[33] Nielsen KR, Steffensen R, Boegsted M, Baech J, Lundbye-Christensen S, Hetland ML, et al Promoter polymorphisms in the chitinase 3-like 1 gene influence the serum concentration of YKL-40 in Danish patients with rheumatoid arthritis and in healthy subjects. Arthritis Res Ther. 2011;13: R109 10.1186/ar3391 21714862 PMC3218924

[34] Bergkvist KS, Nyegaard M, Bøgsted M, Schmitz A, Bødker JS, Rasmussen SM, et al Validation and implementation of a method for microarray gene expression profiling of minor B-cell subpopulations in man. BMC Immunol. 2014;15: 3 10.1186/1471-2172-15-3 24483235 PMC3937209

[35] Gentleman RC, Gentleman RC, Carey VJ, Carey VJ, Bates DM, Bates DM, et al Bioconductor: open software development for computational biology and bioinformatics. Genome Biol. 2004;5: R80 10.1186/gb-2004-5-10-r80 15461798 PMC545600

[36] Dai M, Wang P, Boyd AD, Kostov G, Athey B, Jones EG, et al Evolving gene/transcript definitions significantly alter the interpretation of GeneChip data. Nucleic Acids Res. 2005;33: 1–9. 10.1093/nar/gni179 16284200 PMC1283542

[37] Cerhan JR, Berndt SI, Vijai J, Ghesquières H, McKay J, Wang SS, et al Genome-wide association study identifies multiple susceptibility loci for diffuse large B cell lymphoma. Nat Genet. 2014;46 10.1038/ng.3105 PMC4213349 25261932

[38] Bourke E, Bosisio D, Golay J, Polentarutti N, Mantovani A. The toll-like receptor repertoire of human B lymphocytes: inducible and selective expression of TLR9 and TLR10 in normal and transformed cells. Blood. 2003;102: 956–63. 10.1182/blood-2002-11-3355 12689944

[39] Carvalho A, Cunha C, Almeida AJ, Osório NS, Saraiva M, Teixeira-Coelho M, et al The rs5743836 polymorphism in TLR9 confers a population-based increased risk of non-Hodgkin lymphoma. Genes Immun. 2012;13: 197–201. 10.1038/gene.2011.59 21866115 PMC3876733

[40] Vijai J, Kirchhoff T, Schrader K a, Brown J, Dutra-Clarke AV, Manschreck C, et al Susceptibility loci associated with specific and shared subtypes of lymphoid malignancies. PLoS Genet. 2013;9: e1003220 10.1371/journal.pgen.1003220 23349640 PMC3547842

[41] Gibson TM, Wang SS, Cerhan JR, Maurer MJ, Hartge P, Habermann TM, et al letters Inherited genetic variation and overall survival following follicular lymphoma. 2012; 724–726. 10.1002/ajh.23184 PMC3392094 22473939

[42] Hosgood HD, Purdue MP, Wang SS, Zheng T, Morton LM, Lan Q, et al A pooled analysis of three studies evaluating genetic variation in innate immunity genes and non-Hodgkin lymphoma risk. Br J Haematol. 2011;152: 721–6. 10.1111/j.1365-2141.2010.08518.x 21250972 PMC3253820

[43] Koene HR, Kleijer M, Algra J, Roos D, von dem Borne AE, de Haas M. Fc gammaRIIIa-158V/F polymorphism influences the binding of IgG by natural killer cell Fc gammaRIIIa, independently of the Fc gammaRIIIa-48L/R/H phenotype. Blood. 1997;90: 1109–14. Available: http://www.ncbi.nlm.nih.gov/pubmed/9242542 9242542

[44] Johansen JS, Schultz NA, Jensen BV. Plasma YKL-40: a potential new cancer biomarker? Future Oncol. 2009;5: 1065–82. 10.2217/fon.09.66 19792974

[45] El-Galaly TC, Bilgrau AE, Gaarsdal E, Klausen TW, Pedersen LM, Nielsen KR, Bæch J, Bøgsted M, Dybkær K, Johansen JS, J H. Circulating TNF α and YKL-40 level is associated to remission status following salvage therapy in relapsed non-Hodgkin lymphoma. Leuk Lymphoma. 2015; 10.31 10.3109/10428194.2014.1001984 25563560

[46] Läubli H, Borsig L. Selectins promote tumor metastasis. Semin Cancer Biol. 2010;20: 169–77. 10.1016/j.semcancer.2010.04.005 20452433

[47] Aschebrook-Kilfoy B, Zheng T, Foss F, Ma S, Han X, Lan Q, et al Polymorphisms in immune function genes and non-Hodgkin lymphoma survival. J Cancer Surviv. 2012;6: 102–14. 10.1007/s11764-010-0164-4 22113576 PMC3326600

[48] Takatsu K. Interleukin 5 and B cell differentiation. Cytokine Growth Factor Rev. 1998;9: 25–35. Available: http://www.ncbi.nlm.nih.gov/pubmed/9720754 9720754 10.1016/s1359-6101(97)00034-8

[49] Eurich D, Boas-Knoop S, Morawietz L, Neuhaus R, Somasundaram R, Ruehl M, et al Association of mannose-binding lectin-2 gene polymorphism with the development of hepatitis C-induced hepatocellular carcinoma. Liver Int. 2011;31: 1006–1012. 10.1111/j.1478-3231.2011.02522.x 21733090

[50] Van der Bol JM, de Jong FA, van Schaik RH, Sparreboom A, van Fessem MA, van de Geijn FE, et al Effects of mannose-binding lectin polymorphisms on irinotecan-induced febrile neutropenia. Oncologist. 2010;15: 1063–1072. 10.1634/theoncologist.2010-0033 20930093 PMC3227891

[51] Nielsen KR, Steffensen R, Haunstrup TM, Bødker JS, Dybkær K, Baech J, et al Inherited variation in immune response genes in follicular lymphoma and diffuse large B-cell lymphoma. Leuk Lymphoma. 2015; 1–10. 10.3109/10428194.2015.1058936 26044172

[52] Gascoyne RD, Rosenwald A, Poppema S, Lenz G. Prognostic biomarkers in malignant lymphomas. Leuk Lymphoma. 2010;51 Suppl 1: 11–9. 10.3109/10428194.2010.500046 20658955

[53] Kay NE, Pittner BT. IL-4 biology: impact on normal and leukemic CLL B cells. Leuk Lymphoma. 2003;44: 897–903. 10.1080/1042819031000068007 12854886

[54] Pound JD, Gordon J. Maintenance of human germinal center B cells in vitro. Blood. 1997;89: 919–928. 9028323

[55] Kobayashi N, Nagumo H, Agematsu K. IL-10 enhances B-cell IgE synthesis by promoting differentiation into plasma cells, a process that is inhibited by CD27/CD70 interaction. Clin Exp Immunol. 2002;129: 446–452. 10.1046/j.1365-2249.2002.01932.x 12197885 PMC1906463

[56] Gutiérrez NC, Ocio EM, de Las Rivas J, Maiso P, Delgado M, Fermiñán E, et al Gene expression profiling of B lymphocytes and plasma cells from Waldenström’s macroglobulinemia: comparison with expression patterns of the same cell counterparts from chronic lymphocytic leukemia, multiple myeloma and normal individuals. Leukemia. 2007;21: 541–9. 10.1038/sj.leu.2404520 17252022

[57] Shaffer AL, Young RM, Staudt LM. Pathogenesis of human B cell lymphomas. Annu Rev Immunol. 2012;30: 565–610. 10.1146/annurev-immunol-020711-075027 22224767 PMC7478144

[58] Johnsen HE, Kjeldsen MK, Urup T, Fogd K, Pilgaard L, Boegsted M, et al Cancer stem cells and the cellular hierarchy in haematological malignancies. Eur J Cancer. Elsevier Ltd; 2009;45 Suppl 1: 194–201. 10.1016/S0959-8049(09)70033-4 19775618

[59] Smith AJP, Humphries SE. Cytokine and cytokine receptor gene polymorphisms and their functionality. Cytokine Growth Factor Rev. 2009;20: 43–59. 10.1016/j.cytogfr.2008.11.006 19038572

