# Supplementary material for: Inherited Inflammatory Response Genes Are Associated with B-Cell Non-Hodgkin’s Lymphoma Risk and Survival
Source: PLoS One. 2015 Oct 8;10(10):e0139329. doi: 10.1371/journal.pone.0139329 (PMC4598167; doi:10.1371/journal.pone.0139329)
Supplement: S1 Table — (DOCX) [file pone.0139329.s002.docx]

**S1 Table. Assay information 50SNPs genotyped in 31 genes.**

| **rs nr.** | **SNP** | **Assay ID** | **Forward Primer Sequence** | **Reverse Primer Sequence** | **VIC labelled probe** | **FAM labelled probe** | |
| --- | --- | --- | --- | --- | --- | --- | --- |
| rs12780112 | *MBL2 +4 (P/Q)* | Custom designed | GCACCCAGATTGTAGGACAGA | GTGTGAGAAAACTCAGGGAAGGTTA | AACACATATTTACCGAGCATG | CACATATTTACCAAGCATG |  |
| rs11003125 | *MBL2 -550 G/C* | Custom designed | GGGCCAACGTAGTAAGAAATTTCCA | GGAGTTTGCTTCCCCTTGGT | AAGCCTGTGTAAAAC | AAGCCTGTCTAAAAC |  |
| rs5743815 | *TLR6 T/C* | Custom designed | GCTGGAATTCTTTGGAATCTGGTAGA | AACAGAGTCAGTAAGCATATTTGAAGACAA | CACTATACTCTCAGCCCAAG | CACTATACTCTCAACCCAAG |  |
| rs2234671 | *IL8RA Ex2+860 G/C* | Custom designed | CTCATGAGGACCCAGGTGATC | CCCGGCCGATGTTGTTG | CTCACAGGTCTCCTG | CTCACAGCTCTCCTG |  |
| rs241447 | *TAP-2 A/G* | Custom designed | CGCACAGTGCTGGTGATTG | TTGCCCTCCTGGAGCAC | CGCTGAACTGTCTGCAG | CGCTGAACTGCCTGCAG |  |
| rs78440425 | *CXCR5 G/A* | Custom designed | TTCTCCCTCCTTTGACTGAAACG | GGACTCTTGCTATGAGCTTCCA | CCTCAGCTTCTACCCAGTC | CAGCTTCTGCCCAGTC |  |
| rs80202369 | *CXCR5 G/A* | Custom designed | CGCCTGCTTGCCTGTCT | CTCTCCCATTCTTGGTCTCATAAGG | CACAGGCTAGGATGAG | ACAGGCTGGGATGAG |  |
| rs1800896 | *IL10 -1082 A/G* | C___1747360_10 |  |  |  |  |  |
| rs1800871 | *IL10 -819 C/T* | C___1747362_10 |  |  |  |  |  |
| rs1800872 | *IL10 -592 C/A* | C___1747363_10 |  |  |  |  |  |
| rs1143623 | *IL1B −1464 G/C* | C___1839941_10 |  |  |  |  |  |
| rs16944 | *IL1B -511 G/A* | C___1839943_10 |  |  |  |  |  |
| rs1143627 | *IL-1B -31 T/C* | C___1839944_10 |  |  |  |  |  |
| rs9610 | *IL10RA A/G* | C___2121217_10 |  |  |  |  |  |
| rs1800451 | *MBL2 G57E G/A* | C___2336608_20 |  |  |  |  |  |
| rs1800450 | *MBL2 G54D G/A* | C___2336609_20 |  |  |  |  |  |
| rs5030737 | *MBL2 R52C C/T* | C___2336610_10 |  |  |  |  |  |
| rs1801275 | *IL4RA Q576R A/G* | C___2351160_20 |  |  |  |  |  |
| rs231775 | *CTLA4 +49 A/G* | C___2415786_20 |  |  |  |  |  |
| rs485497 | *IL12A G/A* | C___2423979_10 |  |  |  |  |  |
| rs1058867 | *IL10RB A/G* | C___2443324_20 |  |  |  |  |  |
| rs909253 | *LTα -252 A/G* | C___2451911_10 |  |  |  |  |  |
| rs1805010 | *IL4RA I75V A/G* | C___2769554_10 |  |  |  |  |  |
| rs10987898 | *GALNT12 T/G* | C___3055323_10 |  |  |  |  |  |
| rs2637988 | *IL1R A/G* | C___3133508_10 |  |  |  |  |  |
| rs1799964 | *TNFA -1031 T/C* | C___7514871_10 |  |  |  |  |  |
| rs1800629 | *TNFA -308 G/A* | C___7514879_10 |  |  |  |  |  |
| rs3732379 | *CX3CR1 C/T* | C___7900503_1_ |  |  |  |  |  |
| rs419598 | *IL1RA T/C* | C___8737990_10 |  |  |  |  |  |
| rs1800890 | *IL10 -3575 T/A* | C___8828790_10 |  |  |  |  |  |
| rs1061622 | *TNFRSF1B +676 T/G* | C___8861232_20 |  |  |  |  |  |
| rs1805011 | *IL4RA E400A A/C* | C___8903098_20 |  |  |  |  |  |
| rs1801274 | *FCGR2A C/T* | C___9077561_20 |  |  |  |  |  |
| rs1800796 | *IL-6 -572 G/C* | C__11326893_10 |  |  |  |  |  |
| rs4848306 | *IL-1B -3737* | C__11725735_10 |  |  |  |  |  |
| rs1799724 | *TNFA -857 C/T* | C__11918223_10 |  |  |  |  |  |
| rs5361 | *SELE A/C* | C__11975332_10 |  |  |  |  |  |
| rs2069762 | *IL2 -330 G/T* | C__15859930_10 |  |  |  |  |  |
| rs2104286 | *IL2RA A/G* | C__16095542_10 |  |  |  |  |  |
| rs2243248 | *IL4 1098 T/G* | C__16176227_10 |  |  |  |  |  |
| rs2305742 | *IL12RB1+6193 A/C* | C__16191619_10 |  |  |  |  |  |
| rs2069812 | *IL5 -745 C/T* | C__16274150_10 |  |  |  |  |  |
| rs396991 | *FCGR3A T/G* | C__25815666_10 |  |  |  |  |  |
| rs3775567 | *IRF2 C/T* | C__27499487_10 |  |  |  |  |  |
| rs4950928 | *CHI3L1 -131C/G* | C__27832042_10 |  |  |  |  |  |
| rs7096206 | *MBL2 -221 G/C* | C__27858274_10 |  |  |  |  |  |
| rs6421571 | *CXCR5 C/T* | C__29069283_10 |  |  |  |  |  |
| rs9514828 | *BAFF C/T* | C__29641742_10 |  |  |  |  |  |
| rs16994592 | *TNFSF7 T/C* | C__32993116_10 |  |  |  |  |  |
| rs5743836 | *TLR9 T /C* | C__32645383_10 |  |  |  |  |  |
