# Supplementary material for: Inherited Inflammatory Response Genes Are Associated with B-Cell Non-Hodgkin’s Lymphoma Risk and Survival
Source: PLoS One. 2015 Oct 8;10(10):e0139329. doi: 10.1371/journal.pone.0139329 (PMC4598167; doi:10.1371/journal.pone.0139329)
Supplement: S2 Table — (DOCX) [file pone.0139329.s003.docx]

**S2 Table. Association between SNPs and B-NHL risk**

| **SNP** | **Allele** | **DLBCL** | **Controls** | **OR** |  | **CI** |  | **P value** |
| --- | --- | --- | --- | --- | --- | --- | --- | --- |
| *CXCR5* (rs78440425) | G | 390 | 602 |  |  |  |  |  |
|  | A | 2 | 6 | 0,51 | 0,10 | _ | 2,56 | 0,417 |
| *TAP2* (rs241447) | A | 298 | 440 |  |  |  |  |  |
|  | G | 66 | 160 | 0,61 | 0,44 | _ | 0,84 | 0,003 |
| *IL8RA* (rs2234671) | G | 394 | 572 |  |  |  |  |  |
|  | C | 22 | 36 | 0,89 | 0,51 | _ | 1,53 | 0,667 |
| *TLR6* (rs5743815) | T | 386 | 599 |  |  |  |  |  |
|  | C | 10 | 9 | 1,72 | 0,69 | _ | 4,28 | 0,240 |
| *MBL2* (rs11003125) | G | 254 | 386 |  |  |  |  |  |
|  | C | 142 | 224 | 0,96 | 0,74 | _ | 1,25 | 0,781 |
| *MBL2* (rs12780112) | A | 309 | 476 |  |  |  |  |  |
|  | G | 89 | 126 | 1,09 | 0,80 | _ | 1,48 | 0,590 |
| *TNFSF7* (rs16994592) | T | 343 | 552 |  |  |  |  |  |
|  | C | 29 | 56 | 0,83 | 0,52 | _ | 1,33 | 0,446 |
| *TLR9* (rs5743836) | T | 354 | 523 |  |  |  |  |  |
|  | C | 38 | 85 | 0,66 | 0,44 | _ | 0,99 | 0,045 |
| *BAFF* (rs9514828) | C | 204 | 311 |  |  |  |  |  |
|  | T | 196 | 299 | 0,99 | 0,78 | _ | 1,29 | 0,996 |
| *CXCR5* (rs6421571) | C | 291 | 473 |  |  |  |  |  |
|  | T | 77 | 133 | 0,94 | 0,69 | _ | 1,29 | 0,707 |
| *MBL2* (rs7096206) | C | 318 | 455 |  |  |  |  |  |
|  | G | 96 | 153 | 0,90 | 0,67 | _ | 1,20 | 0,470 |
| *CHI3L1* (rs4950928) | C | 333 | 477 |  |  |  |  |  |
|  | G | 71 | 123 | 0,83 | 0,60 | _ | 1,14 | 0,250 |
| *IRF2* (rs3775567) | C | 397 | 572 |  |  |  |  |  |
|  | T | 17 | 34 | 0,72 | 0,40 | _ | 1,31 | 0,281 |
| *FCGR3A* (rs396991) | T | 283 | 424 |  |  |  |  |  |
|  | G | 113 | 182 | 0,93 | 0,70 | _ | 1,23 | 0,611 |
| *IL5* (rs2069812) | C | 272 | 439 |  |  |  |  |  |
|  | T | 110 | 171 | 1,04 | 0,78 | _ | 1,38 | 0,795 |
| *IL12RB1* (rs2305742) | A | 323 | 488 |  |  |  |  |  |
|  | C | 89 | 122 | 1,10 | 0,81 | _ | 1,50 | 0,535 |
| *IL4* (rs2243248) | T | 364 | 575 |  |  |  |  |  |
|  | G | 36 | 35 | 1,62 | 1,00 | _ | 2,63 | 0,049 |
| *IL2RA* (rs2104286) | A | 310 | 427 |  |  |  |  |  |
|  | G | 98 | 183 | 0,74 | 0,55 | _ | 0,98 | 0,037 |
| *IL2* (rs2069762) | G | 221 | 441 |  |  |  |  |  |
|  | T | 99 | 167 | 1,18 | 0,88 | _ | 1,59 | 0,267 |
| *SELE* (rs5361) | A | 346 | 531 |  |  |  |  |  |
|  | C | 44 | 77 | 0,88 | 0,59 | _ | 1,30 | 0,514 |
| *TNFA* (rs1799724) | C | 370 | 559 |  |  |  |  |  |
|  | T | 6 | 51 | 0,18 | 0,08 | _ | 0,42 | 0,000 |
| *IL1B* (rs419598) | G | 219 | 351 |  |  |  |  |  |
|  | A | 157 | 259 | 0,97 | 0,75 | _ | 1,26 | 0,828 |
| *IL6* (rs1800796) | G | 399 | 589 |  |  |  |  |  |
|  | C | 15 | 21 | 1,05 | 0,54 | _ | 2,07 | 0,878 |
| *FCGR2A* (rs1801274) | C | 226 | 310 |  |  |  |  |  |
|  | T | 170 | 300 | 1,37 | 1,07 | _ | 1,77 | 0,014 |
| *IL4R* (rs1805011) | A | 353 | 540 |  |  |  |  |  |
|  | C | 53 | 64 | 1,27 | 0,86 | _ | 1,87 | 0,232 |
| *TNFRSF1B* (rs1061622) | T | 291 | 459 |  |  |  |  |  |
|  | G | 99 | 149 | 1,05 | 0,78 | _ | 1,41 | 0,754 |
| *IL10* (rs1800890) | T | 224 | 358 |  |  |  |  |  |
|  | A | 144 | 250 | 0,92 | 0,71 | _ | 1,20 | 0,540 |
| *IL1RA* (rs419598) | T | 223 | 452 |  |  |  |  |  |
|  | C | 85 | 156 | 1,06 | 0,78 | _ | 1,44 | 0,724 |
| *CX3CR1* (rs373379) | C | 295 | 447 |  |  |  |  |  |
|  | T | 115 | 163 | 1,07 | 0,81 | _ | 1,41 | 0,641 |
| *TNFA* (rs1800629) | G | 318 | 509 |  |  |  |  |  |
|  | A | 92 | 101 | 1,46 | 1,06 | _ | 2,00 | 0,019 |
| *TNFA* (rs1799964) | T | 301 | 474 |  |  |  |  |  |
|  | C | 41 | 134 | 0,48 | 0,33 | _ | 0,70 | 0,000 |
| *IL1R (*rs2637988) | A | 243 | 390 |  |  |  |  |  |
|  | G | 161 | 220 | 1,175 | 0,91 | _ | 1,52 | 0,223 |
| *GALNT12* (rs10987898) | T | 273 | 424 |  |  |  |  |  |
|  | G | 105 | 170 | 0,96 | 0,72 | _ | 1,28 | 0,776 |
| *IL4R* (rs1805010) | A | 224 | 325 |  |  |  |  |  |
|  | G | 180 | 285 | 0,92 | 0,71 | _ | 1,18 | 0,498 |
| *CXCR5* (rs80202369) | G | 394 | 602 |  |  |  |  |  |
|  | A | 0 | 0 | - |  |  | - | - |
| *LTA* (rs909253) | T | 256 | 399 |  |  |  |  |  |
|  | C | 154 | 211 | 1,14 | 0,88 | _ | 1,48 | 0,332 |
| *IL10RB* (rs1058867) | A | 256 | 338 |  |  |  |  |  |
|  | G | 138 | 266 | 0,68 | 0,53 | _ | 0,89 | 0,005 |
| *IL12A* (rs485497) | G | 216 | 313 |  |  |  |  |  |
|  | A | 178 | 297 | 0,87 | 0,67 | _ | 1,12 | 0,277 |
| *CTLA4* (rs231775) | A | 229 | 343 |  |  |  |  |  |
|  | G | 171 | 265 | 0,97 | 0,75 | _ | 1,25 | 0,793 |
| *IL4RA* (rs1801275) | A | 304 | 485 |  |  |  |  |  |
|  | G | 80 | 123 | 1,04 | 0,76 | _ | 1,42 | 0,819 |
| *MBL2* (rs5030737) | C | 373 | 557 |  |  |  |  |  |
|  | T | 19 | 41 | 0,69 | 0,40 | _ | 1,21 | 0,197 |
| *MBL2* (rs1800450) | G | 351 | 527 |  |  |  |  |  |
|  | A | 57 | 77 | 1,11 | 0,77 | _ | 1,61 | 0,574 |
| *MBL2* (rs1800451) | G | 346 | 599 |  |  |  |  |  |
|  | A | 0 | 7 | - |  |  | - | - |
| *IL10RA* (rs9610) | A | 238 | 346 |  |  |  |  |  |
|  | G | 170 | 254 | 0,97 | 0,75 | _ | 1,26 | 0,833 |
| *IL1B* (rs1143627) | T | 273 | 377 |  |  |  |  |  |
|  | C | 131 | 231 | 0,78 | 0,60 | _ | 1,02 | 0,071 |
| *IL1B* (rs16944) | G | 273 | 374 |  |  |  |  |  |
|  | A | 135 | 230 | 0,80 | 0,62 | _ | 1,05 | 0,105 |
| *IL1B* (rs1143623) | G | 298 | 424 |  |  |  |  |  |
|  | C | 106 | 184 | 0,82 | 0,62 | _ | 1,09 | 0,166 |
| *IL10* (rs1800872) | C | 330 | 475 |  |  |  |  |  |
|  | A | 86 | 133 | 0,93 | 0,69 | _ | 1,26 | 0,645 |
| *IL10* (rs1800871) | C | 266 | 474 |  |  |  |  |  |
|  | T | 78 | 134 | 1,04 | 0,76 | _ | 1,42 | 0,821 |
| *IL10 (*rs1800896) | G | 225 | 318 |  |  |  |  |  |
|  | A | 187 | 284 | 0,93 | 0,72 | _ | 1,20 | 0,575 |
