# Supplementary material for: Inherited Inflammatory Response Genes Are Associated with B-Cell Non-Hodgkin’s Lymphoma Risk and Survival
Source: PLoS One. 2015 Oct 8;10(10):e0139329. doi: 10.1371/journal.pone.0139329 (PMC4598167; doi:10.1371/journal.pone.0139329)
Supplement: S4 Table — (DOCX) [file pone.0139329.s005.docx]

**S4 Table. Association between SNPs and risk of FL**

| **SNP** | **Allele** | **DLBCL** | **Controls** | **OR** |  | **CI** |  | **P value** |
| --- | --- | --- | --- | --- | --- | --- | --- | --- |
| *CXCR5* (rs80202369) | G | 264 | 602 |  |  |  |  |  |
|  | A | 0 | 0 | - |  | _ |  | - |
| *CXCR5* (rs78440425) | G | 263 | 602 |  |  |  |  |  |
|  | A | 3 | 6 | 1,14 | 0,28 | _ | 4,61 | 0,849 |
| *TAP2* (rs241447) | A | 181 | 440 |  |  |  |  |  |
|  | G | 61 | 160 | 0,93 | 0,66 | _ | 1,30 | 0,663 |
| *IL8RA* (rs2234671) | G | 259 | 572 |  |  |  |  |  |
|  | C | 17 | 36 | 1,05 | 0,58 | _ | 1,89 | 0,890 |
| *TLR6* (rs5743815) | T | 265 | 599 |  |  |  |  |  |
|  | C | 1 | 9 | 0,25 | 0,03 | _ | 1,99 | 0,191 |
| *MBL2* (rs11003125) | G | 165 | 386 |  |  |  |  |  |
|  | C | 101 | 224 | 1,05 | 0,78 | _ | 1,42 | 0,725 |
| *MBL2* (rs12780112) | A | 202 | 476 |  |  |  |  |  |
|  | G | 66 | 126 | 1,23 | 0,88 | _ | 1,73 | 0,225 |
| *TNFSF7* (rs16994592) | T | 241 | 552 |  |  |  |  |  |
|  | C | 15 | 56 | 0,61 | 0,34 | _ | 1,11 | 0,104 |
| *TLR9* (rs5743836) | T | 239 | 523 |  |  |  |  |  |
|  | C | 27 | 85 | 0,70 | 0,44 | _ | 1,10 | 0,121 |
| *BAFF* (rs9514828) | C | 142 | 311 |  |  |  |  |  |
|  | T | 130 | 299 | 1,14 | 0,85 | _ | 1,51 | 0,382 |
| *CXCR5* (rs6421571) | C | 196 | 473 |  |  |  |  |  |
|  | T | 46 | 133 | 0,83 | 0,57 | _ | 1,21 | 0,344 |
| *MBL2* (rs7096206) | C | 222 | 455 |  |  |  |  |  |
|  | G | 54 | 153 | 0,72 | 0,51 | _ | 1,03 | 0,069 |
| *CHI3L1* (rs4950928) | C | 221 | 477 |  |  |  |  |  |
|  | G | 53 | 123 | 0,93 | 0,65 | _ | 1,33 | 0,692 |
| *IRF2* (rs3775567) | C | 253 | 572 |  |  |  |  |  |
|  | T | 15 | 34 | 0,99 | 0,53 | _ | 1,86 | 0,994 |
| *FCGR3A* (rs396991) | T | 176 | 424 |  |  |  |  |  |
|  | G | 88 | 182 | 1,16 | 0,85 | _ | 1,59 | 0,334 |
| *IL5* (rs2069812) | C | 171 | 439 |  |  |  |  |  |
|  | T | 81 | 171 | 1,22 | 0,88 | _ | 1,67 | 0,228 |
| *IL12RB1* (rs2305742) | A | 217 | 488 |  |  |  |  |  |
|  | C | 57 | 122 | 1,05 | 0,74 | _ | 1,50 | 0,784 |
| *IL4* (rs2243248) | T | 253 | 575 |  |  |  |  |  |
|  | G | 15 | 35 | 0,97 | 0,52 | _ | 1,82 | 0,934 |
| *IL2RA* (rs2104286) | A | 198 | 427 |  |  |  |  |  |
|  | G | 74 | 183 | 0,87 | 0,63 | _ | 1,20 | 0,399 |
| *IL2* (rs2069762) | G | 163 | 441 |  |  |  |  |  |
|  | T | 49 | 167 | 0,79 | 0,55 | _ | 1,14 | 0,216 |
| *SELE* (rs5361) | A | 228 | 531 |  |  |  |  |  |
|  | C | 34 | 77 | 1,03 | 0,67 | _ | 1,58 | 0,899 |
| *TNFA* (rs1799724) | C | 244 | 559 |  |  |  |  |  |
|  | T | 4 | 51 | 0,18 | 0,06 | _ | 0,50 | 0,001 |
| *IL1B* (rs419598) | G | 141 | 351 |  |  |  |  |  |
|  | A | 119 | 259 | 1,14 | 0,85 | _ | 1,53 | 0,367 |
| *IL6* (rs1800796) | G | 256 | 589 |  |  |  |  |  |
|  | C | 16 | 21 | 1,75 | 0,90 | _ | 3,41 | 0,099 |
| *FCGR2A* (rs1801274) | C | 156 | 310 |  |  |  |  |  |
|  | T | 114 | 300 | 0,76 | 0,57 | _ | 1,01 | 0,057 |
| *IL4R* (rs1805011) | A | 239 | 540 |  |  |  |  |  |
|  | C | 29 | 64 | 1,02 | 0,64 | _ | 1,63 | 0,921 |
| *TNFRSF1B* (rs1061622) | T | 200 | 459 |  |  |  |  |  |
|  | G | 58 | 149 | 0,89 | 0,63 | _ | 1,26 | 0,523 |
| *IL10* (rs1800890) | T | 151 | 358 |  |  |  |  |  |
|  | A | 107 | 250 | 1,01 | 0,75 | _ | 1,36 | 0,923 |
| *IL1RA* (rs419598) | T | 167 | 452 |  |  |  |  |  |
|  | C | 45 | 156 | 0,78 | 0,54 | _ | 1,14 | 0,197 |
| *CX3CR1* (rs373379) | C | 196 | 447 |  |  |  |  |  |
|  | T | 68 | 163 | 0,95 | 0,68 | _ | 1,32 | 0,767 |
| *TNFA* (rs1800629) | G | 221 | 509 |  |  |  |  |  |
|  | A | 51 | 101 | 1,16 | 0,80 | _ | 1,69 | 0,426 |
| *TNFA* (rs1799964) | T | 189 | 474 |  |  |  |  |  |
|  | C | 27 | 134 | 0,50 | 0,32 | _ | 0,79 | 0,003 |
| *IL1R (*rs2637988) | A | 164 | 390 |  |  |  |  |  |
|  | G | 104 | 220 | 1,12 | 0,84 | _ | 1,51 | 0,438 |
| *GALNT12* (rs10987898) | T | 192 | 424 |  |  |  |  |  |
|  | G | 54 | 170 | 0,70 | 0,49 | _ | 1,00 | 0,047 |
| *IL4R* (rs1805010) | A | 152 | 325 |  |  |  |  |  |
|  | G | 114 | 285 | 0,86 | 0,64 | _ | 1,14 | 0,291 |
| *LTA* (rs909253) | T | 179 | 399 |  |  |  |  |  |
|  | C | 93 | 211 | 0,98 | 0,73 | _ | 1,33 | 0,908 |
| *IL10RB* (rs1058867) | A | 165 | 338 |  |  |  |  |  |
|  | G | 99 | 266 | 0,76 | 0,57 | _ | 1,03 | 0,073 |
| *IL12A* (rs485497) | G | 154 | 313 |  |  |  |  |  |
|  | A | 104 | 297 | 0,71 | 0,53 | _ | 0,96 | 0,024 |
| *CTLA4* (rs231775) | A | 160 | 343 |  |  |  |  |  |
|  | G | 106 | 265 | 0,86 | 0,64 | _ | 1,15 | 0,304 |
| *IL4RA* (rs1801275) | A | 209 | 485 |  |  |  |  |  |
|  | G | 51 | 123 | 0,96 | 0,67 | _ | 1,39 | 0,836 |
| *MBL2* (rs5030737) | C | 246 | 557 |  |  |  |  |  |
|  | T | 16 | 41 | 0,88 | 0,49 | _ | 1,61 | 0,684 |
| *MBL2* (rs1800450) | G | 228 | 527 |  |  |  |  |  |
|  | A | 38 | 77 | 1,14 | 0,75 | _ | 1,73 | 0,538 |
| *MBL2* (rs1800451) | G | 234 | 599 |  |  |  |  |  |
|  | A | 0 | 7 | - |  | _ |  | - |
| *IL10RA* (rs9610) | A | 166 | 346 |  |  |  |  |  |
|  | G | 106 | 254 | 0,87 | 0,65 | _ | 1,17 | 0,350 |
| *IL1B* (rs1143627) | T | 193 | 377 |  |  |  |  |  |
|  | C | 77 | 231 | 0,65 | 0,48 | _ | 0,89 | 0,007 |
| *IL1B* (rs16944) | G | 191 | 374 |  |  |  |  |  |
|  | A | 79 | 230 | 0,67 | 0,49 | _ | 0,92 | 0,012 |
| *IL1B* (rs1143623) | G | 205 | 424 |  |  |  |  |  |
|  | C | 67 | 184 | 0,75 | 0,54 | _ | 1,04 | 0,088 |
| *IL10* (rs1800872) | C | 218 | 475 |  |  |  |  |  |
|  | A | 58 | 133 | 0,95 | 0,67 | _ | 1,35 | 0,773 |
| *IL10* (rs1800871) | C | 186 | 474 |  |  |  |  |  |
|  | T | 42 | 134 | 0,80 | 0,54 | _ | 1,17 | 0,254 |
| *IL10 (*rs1800896) | G | 137 | 318 |  |  |  |  |  |
|  | A | 133 | 284 | 1,15 | 0,87 | _ | 1,54 | 0,330 |
