# Supplementary material for: Inherited Inflammatory Response Genes Are Associated with B-Cell Non-Hodgkin’s Lymphoma Risk and Survival
Source: PLoS One. 2015 Oct 8;10(10):e0139329. doi: 10.1371/journal.pone.0139329 (PMC4598167; doi:10.1371/journal.pone.0139329)
Supplement: S6 Table — (DOCX) [file pone.0139329.s007.docx]

**S6 Table. Assoication between SNPs and 10 year overall survival for DLBCL. The model is adjusted for sex, IPI and treatment.**

| **SNP** | **Genotype** | **HR** |  | **CI** |  | **P value** |
| --- | --- | --- | --- | --- | --- | --- |
| *CXCR5* (rs78440425) | G A | 1,00 |  |  |  |  |
|  | G G | 0,68 | 0,09 | _ | 4,97 | 0,702 |
| *TAP2* (rs241447) | A A | 1,00 |  |  |  |  |
|  | A G | 0,88 | 0,56 | _ | 1,38 | 0,572 |
|  | G G | 3,17 | 1,21 | _ | 8,32 | 0,019 |
| *IL8RA* (rs2234671) | C C | 1,00 |  |  |  |  |
|  | C G | 1,17 | 0,61 | _ | 2,27 | 0,633 |
|  | G G | 0,89 | 0,21 | _ | 3,72 | 0,877 |
| *TLR6* (rs5743815) | C C | 1,00 |  |  |  |  |
|  | C T | 0,59 | 0,19 | _ | 1,88 | 0,374 |
|  | T T | 0,00 | 0,00 |  |  | 1,000 |
| *MBL2* (rs11003125) | C C | 1,00 |  |  |  |  |
|  | C G | 0,74 | 0,50 | _ | 1,10 | 0,133 |
|  | G G | 0,70 | 0,41 | _ | 1,20 | 0,194 |
| rs12780112 | A A | 1,00 |  |  |  |  |
|  | A G | 0,70 | 0,46 | _ | 1,06 | 0,089 |
|  | G G | 1,47 | 0,66 | _ | 3,27 | 0,347 |
| *TNFSF7* (rs16994592) | C C | 1,00 |  |  |  |  |
|  | C T | 1,04 | 0,60 | _ | 1,78 | 0,893 |
|  | T T | 20,79 | 2,49 | _ | 173,63 | 0,005 |
| *TLR9* (rs5743836) | T T | 1,00 |  |  |  |  |
|  | T C | 0,95 | 0,61 | _ | 1,49 | 0,830 |
|  | C C | 1,00 |  |  |  |  |
| *BAFF* (rs9514828) | C C | 0,94 | 0,60 | _ | 1,46 | 0,768 |
|  | C T | 1,09 | 0,66 | _ | 1,80 | 0,725 |
| *CXCR5* (rs6421571) | C C | 1,00 |  |  |  |  |
|  | C T | 0,86 | 0,56 | _ | 1,32 | 0,487 |
|  | T T | 1,48 | 0,66 | _ | 3,28 | 0,339 |
| *MBL2* (rs7096206) | C C | 1,00 |  |  |  |  |
|  | C G | 1,47 | 1,02 | _ | 2,13 | 0,041 |
|  | G G | 1,15 | 0,52 | _ | 2,57 | 0,727 |
| *CHI3L1* (rs4950928) | C C | 1,00 |  |  |  |  |
|  | C G | 0,98 | 0,66 | _ | 1,44 | 0,914 |
|  | G G | 0,55 | 0,17 | _ | 1,80 | 0,321 |
| *IRF2* (rs3775567) | C C | 1,00 |  |  |  |  |
|  | C T | 0,92 | 0,44 | _ | 1,90 | 0,813 |
|  | T T | 3,69 | 0,49 | _ | 27,80 | 0,206 |
| *FCGR3A* (rs396991) | G G | 1,00 |  |  |  |  |
|  | G T | 1,03 | 0,70 | _ | 1,51 | 0,880 |
|  | T T | 0,73 | 0,35 | _ | 1,53 | 0,406 |
| *IL5* (rs2069812) | C C | 1,00 |  |  |  |  |
|  | T C | 1,06 | 0,70 | _ | 1,61 | 0,778 |
|  | T T | 1,94 | 1,12 | _ | 3,39 | 0,019 |
| *IL12RB1* (rs2305742) | A A | 1,00 |  |  |  |  |
|  | A C | 0,88 | 0,59 | _ | 1,32 | 0,535 |
|  | C C | 1,59 | 0,81 | _ | 3,13 | 0,182 |
| *IL4* (rs2243248) | G G | 1,00 |  |  |  |  |
|  | G T | 1,11 | 0,65 | _ | 1,88 | 0,708 |
|  | T T | 0,63 | 0,19 | _ | 2,12 | 0,458 |
| *IL2RA* (rs2104286) | A A | 1,00 |  |  |  |  |
|  | G A | 0,89 | 0,61 | _ | 1,30 | 0,533 |
|  | G G | 0,46 | 0,19 | _ | 1,08 | 0,073 |
| *IL2* (rs2069762) | G G | 1,00 |  |  |  |  |
|  | G T | 0,90 | 0,56 | _ | 1,43 | 0,654 |
|  | T T | 1,78 | 0,92 | _ | 3,42 | 0,085 |
| *SELE* (rs5361) | A A | 1,00 |  |  |  |  |
|  | C A | 0,73 | 0,45 | _ | 1,18 | 0,201 |
|  | C C | 0,00 | 0,00 |  |  | 1,000 |
| *TNFA* (rs1799724) | C C | 1,00 |  |  |  |  |
|  | C T | 1,39 | 0,56 | _ | 3,48 | 0,476 |
| *IL1B* (rs419598) | A A | 1,00 |  |  |  |  |
|  | A G | 1,05 | 0,68 | _ | 1,61 | 0,825 |
|  | G G | 0,69 | 0,38 | _ | 1,27 | 0,238 |
| *IL6* (rs1800796) | C C | 1,00 |  |  |  |  |
|  | C G | 1,80 | 0,89 | _ | 3,62 | 0,100 |
|  | G G | 0,00 | 0,00 |  |  | 1,000 |
| *FCGR2A* (rs1801274) | C C | 1,00 |  |  |  |  |
|  | T C | 1,34 | 0,82 | _ | 2,18 | 0,241 |
|  | T T | 0,69 | 0,40 | _ | 1,18 | 0,173 |
| *IL4R* (rs1805011) | A A | 1,00 |  |  |  |  |
|  | A C | 1,25 | 0,82 | _ | 1,90 | 0,303 |
|  | C C | 1,05 | 0,36 | _ | 3,03 | 0,928 |
| *TNFRSF1B* (rs1061622) | G G | 1,00 |  |  |  |  |
|  | G T | 1,34 | 0,89 | _ | 2,03 | 0,160 |
|  | T T | 1,16 | 0,65 | _ | 2,08 | 0,615 |
| *IL10* (rs1800890) | A A | 1,00 |  |  |  |  |
|  | T A | 1,11 | 0,74 | _ | 1,67 | 0,622 |
|  | T T | 0,71 | 0,41 | _ | 1,24 | 0,231 |
| *IL1RA* (rs419598) | C C | 1,00 |  |  |  |  |
|  | C T | 1,29 | 0,84 | _ | 1,97 | 0,247 |
|  | T T | 0,90 | 0,36 | _ | 2,29 | 0,830 |
| *CX3CR1* (rs373379) | C C | 1,00 |  |  |  |  |
|  | C T | 0,86 | 0,59 | _ | 1,27 | 0,456 |
|  | T T | 2,01 | 1,13 | _ | 3,57 | 0,018 |
| *TNFA* (rs1800629) | A A | 1,00 |  |  |  |  |
|  | A G | 1,17 | 0,80 | _ | 1,71 | 0,417 |
|  | G G | 1,02 | 0,51 | _ | 2,05 | 0,946 |
| *TNFA* (rs1799964) | C C | 1,00 |  |  |  |  |
|  | C T | 1,62 | 1,00 | _ | 2,63 | 0,052 |
|  | T T | 4,54 | 0,52 | _ | 39,29 | 0,170 |
| *IL1R (*rs2637988) | A A | 1,00 |  |  |  |  |
|  | A G | 0,95 | 0,63 | _ | 1,42 | 0,785 |
|  | G G | 1,46 | 0,84 | _ | 2,54 | 0,175 |
| *GALNT12* (rs10987898) | G G | 1,00 |  |  |  |  |
|  | G T | 1,33 | 0,87 | _ | 2,02 | 0,191 |
|  | T T | 1,16 | 0,65 | _ | 2,06 | 0,622 |
| *IL4R* (rs1805010) | A A | 1,00 |  |  |  |  |
|  | A G | 1,33 | 0,88 | _ | 2,02 | 0,172 |
|  | G G | 1,15 | 0,68 | _ | 1,94 | 0,597 |
| *LTA* (rs909253) | C C | 1,00 |  |  |  |  |
|  | T C | 1,23 | 0,82 | _ | 1,84 | 0,318 |
|  | T T | 0,88 | 0,51 | _ | 1,51 | 0,633 |
| *IL10RB* (rs1058867) | A A | 1,00 |  |  |  |  |
|  | A G | 1,01 | 0,68 | _ | 1,50 | 0,975 |
|  | G G | 1,31 | 0,72 | _ | 2,37 | 0,375 |
| *IL12A* (rs485497) | A A | 1,00 |  |  |  |  |
|  | A G | 1,04 | 0,67 | _ | 1,60 | 0,874 |
|  | G G | 1,76 | 1,06 | _ | 2,90 | 0,028 |
| *CTLA4* (rs231775) | A A | 1,00 |  |  |  |  |
|  | A G | 0,92 | 0,62 | _ | 1,36 | 0,677 |
|  | G G | 0,71 | 0,41 | _ | 1,21 | 0,203 |
| *IL4RA* (rs1801275) | A A | 1,00 |  |  |  |  |
|  | A G | 1,17 | 0,78 | _ | 1,75 | 0,450 |
|  | G G | 0,72 | 0,24 | _ | 2,12 | 0,553 |
| *MBL2* (rs5030737) | C C | 1,00 |  |  |  |  |
|  | T C | 1,29 | 0,71 | _ | 2,33 | 0,403 |
| *MBL2* (rs1800450) | A A | 1,00 |  |  |  |  |
|  | G A | 1,23 | 0,77 | _ | 1,96 | 0,376 |
|  | G G | 0,88 | 0,35 | _ | 2,19 | 0,779 |
| *MBL2* (rs1800451) | G G | 1,00 |  | - |  | - |
| *IL10RA* (rs9610) | A A | 1,00 |  |  |  |  |
|  | A G | 1,05 | 0,70 | _ | 1,59 | 0,806 |
|  | G G | 1,29 | 0,76 | _ | 2,17 | 0,344 |
| *IL1B* (rs1143627) | C C | 1,00 |  |  |  |  |
|  | C T | 1,29 | 0,89 | _ | 1,87 | 0,181 |
|  | T T | 0,54 | 0,28 | _ | 1,07 | 0,078 |
| *IL1B* (rs16944) | A A | 1,00 |  |  |  |  |
|  | G A | 1,34 | 0,92 | _ | 1,94 | 0,126 |
|  | G G | 0,62 | 0,31 | _ | 1,22 | 0,165 |
| *IL1B* (rs1143623) | C C | 1,00 |  |  |  |  |
|  | G C | 1,33 | 0,93 | _ | 1,91 | 0,122 |
|  | G G | 0,45 | 0,16 | _ | 1,23 | 0,120 |
| IL10 rs1800871 | C C | 1,00 |  |  |  |  |
|  | T C | 1,02 | 0,65 | _ | 1,60 | 0,942 |
|  | T T | 0,75 | 0,33 | _ | 1,68 | 0,482 |
| IL10 rs1800896 | A A | 1,00 |  |  |  |  |
|  | A G | 1,29 | 0,86 | _ | 1,94 | 0,220 |
|  | G G | 1,07 | 0,64 | _ | 1,78 | 0,803 |
