# Supplementary material for: Inherited Inflammatory Response Genes Are Associated with B-Cell Non-Hodgkin’s Lymphoma Risk and Survival
Source: PLoS One. 2015 Oct 8;10(10):e0139329. doi: 10.1371/journal.pone.0139329 (PMC4598167; doi:10.1371/journal.pone.0139329)
Supplement: S7 Table — (DOCX) [file pone.0139329.s008.docx]

**S7 Table. Assoication between SNPs and 10 year overall survival for FL. The model is adjusted for sex, IPI and treatment.**

| **SNP** | **Genotype** | **HR** |  | **CI** |  | **P value** |
| --- | --- | --- | --- | --- | --- | --- |
| *CXCR5* (rs80202369) | G G | 1,00 |  | _ |  | - |
| *TAP2* (rs241447) | A A | 1,00 |  |  |  |  |
|  | A G | 1,04 | 0,58 | _ | 1,85 | 0,900 |
|  | G G | 1,65 | 0,47 | _ | 5,80 | 0,438 |
| *IL8RA* (rs2234671) | C C | 1,00 |  |  |  |  |
|  | C G | 0,77 | 0,32 | _ | 1,84 | 0,559 |
| *TLR6* (rs5743815) | C C | 1,00 |  |  |  |  |
|  | C T | 3,67 | 0,47 | _ | 28,74 | 0,216 |
| *MBL2* (rs11003125) | C C | 1,00 |  |  |  |  |
|  | C G | 0,99 | 0,57 | _ | 1,72 | 0,975 |
|  | G G | 1,33 | 0,53 | _ | 3,35 | 0,540 |
| rs12780112 | A A | 1,00 |  |  |  |  |
|  | A G | 0,94 | 0,51 | _ | 1,74 | 0,854 |
|  | G G | 0,70 | 0,29 | _ | 1,68 | 0,422 |
| *TNFSF7* (rs16994592) | C C | 1,00 |  |  |  |  |
|  | C T | 2,07 | 0,85 | _ | 5,03 | 0,110 |
|  | T T | 2,77 | 0,34 | _ | 22,26 | 0,339 |
| *TLR9* (rs5743836) | T C | 1,00 |  |  |  |  |
|  | T T | 1,01 | 0,52 | _ | 1,95 | 0,984 |
| *BAFF* (rs9514828) | C C | 1,00 |  |  |  |  |
|  | C T | 1,06 | 0,53 | _ | 2,13 | 0,867 |
|  | T T | 0,57 | 0,24 | _ | 1,32 | 0,187 |
| *CXCR5* (rs6421571) | C C | 1,00 |  |  |  |  |
|  | C T | 0,94 | 0,49 | _ | 1,80 | 0,853 |
|  | T T | 0,89 | 0,21 | _ | 3,78 | 0,874 |
| *MBL2* (rs7096206) | C C | 1,00 |  |  |  |  |
|  | C G | 0,79 | 0,42 | _ | 1,49 | 0,471 |
|  | G G | 1,54 | 0,51 | _ | 4,62 | 0,442 |
| *CHI3L1* (rs4950928) | C C | 1,00 |  |  |  |  |
|  | C G | 2,04 | 1,17 | _ | 3,54 | 0,012 |
|  | G G | 0,33 | 0,09 | _ | 1,27 | 0,106 |
| *IRF2* (rs3775567) | C C | 1,00 |  |  |  |  |
|  | C T | 0,58 | 0,20 | _ | 1,63 | 0,299 |
| *FCGR3A* (rs396991) | G G | 1,00 |  |  |  |  |
|  | G T | 1,54 | 0,89 | _ | 2,69 | 0,125 |
|  | T T | 1,37 | 0,53 | _ | 3,53 | 0,513 |
| *IL5* (rs2069812) | C C | 1,00 |  |  |  |  |
|  | T C | 0,85 | 0,49 | _ | 1,50 | 0,582 |
|  | T T | 0,49 | 0,20 | _ | 1,23 | 0,129 |
| *IL12RB1* (rs2305742) | A A | 1,00 |  |  |  |  |
|  | A C | 0,80 | 0,42 | _ | 1,51 | 0,484 |
|  | C C | 1,53 | 0,59 | _ | 3,97 | 0,379 |
| *IL4* (rs2243248) | G G | 1,00 |  |  |  |  |
|  | G T | 1,02 | 0,45 | _ | 2,31 | 0,970 |
|  | T T | 5,34 | 0,68 | _ | 42,12 | 0,112 |
| *IL2RA* (rs2104286) | A A | 1,00 |  |  |  |  |
|  | G A | 0,68 | 0,38 | _ | 1,21 | 0,186 |
|  | G G | 0,59 | 0,21 | _ | 1,68 | 0,322 |
| *IL2* (rs2069762) | G G | 1,00 |  |  |  |  |
|  | G T | 1,15 | 0,57 | _ | 2,32 | 0,695 |
|  | T T | 0,76 | 0,17 | _ | 3,36 | 0,714 |
| *SELE* (rs5361) | A A | 1,00 |  |  |  |  |
|  | C A | 1,21 | 0,59 | _ | 2,44 | 0,604 |
|  | C C | 0,84 | 0,20 | _ | 3,50 | 0,809 |
| *TNFA* (rs1799724) | C C | 1,00 |  |  |  |  |
|  | C T | 0,79 | 0,16 | _ | 4,04 | 0,781 |
| *IL1B* (rs419598) | A A | 1,00 |  |  |  |  |
|  | A G | 1,12 | 0,61 | _ | 2,05 | 0,713 |
|  | G G | 0,56 | 0,27 | _ | 1,19 | 0,132 |
| *IL6* (rs1800796) | C C | 1,00 |  |  |  |  |
|  | C G | 1,32 | 0,63 | _ | 2,76 | 0,460 |
| *FCGR2A* (rs1801274) | C C | 1,00 |  |  |  |  |
|  | T C | 0,90 | 0,50 | _ | 1,63 | 0,726 |
|  | T T | 1,81 | 0,88 | _ | 3,70 | 0,106 |
| *IL4R* (rs1805011) | A A | 1,00 |  |  |  |  |
|  | A C | 1,13 | 0,59 | _ | 2,16 | 0,711 |
|  | C C | 0,00 | 0,00 | _ |  | 1,000 |
| *TNFRSF1B* (rs1061622) | G G | 1,00 |  |  |  |  |
|  | G T | 1,43 | 0,83 | _ | 2,45 | 0,196 |
|  | T T | 0,54 | 0,07 | _ | 4,06 | 0,548 |
| *IL10* (rs1800890) | A A | 1,00 |  |  |  |  |
|  | T A | 0,71 | 0,39 | _ | 1,31 | 0,277 |
|  | T T | 0,90 | 0,44 | _ | 1,86 | 0,778 |
| *IL1RA* (rs419598) | C C | 1,00 |  |  |  |  |
|  | C T | 1,90 | 0,98 | _ | 3,68 | 0,056 |
|  | T T | 1,27 | 0,46 | _ | 3,54 | 0,642 |
| *CX3CR1* (rs373379) | C C | 1,00 |  |  |  |  |
|  | C T | 0,90 | 0,51 | _ | 1,59 | 0,723 |
|  | T T | 4,21 | 1,67 | _ | 10,61 | 0,002 |
| *TNFA* (rs1800629) | A A | 1,00 |  |  |  |  |
|  | A G | 1,46 | 0,82 | _ | 2,62 | 0,202 |
|  | G G | 1,93 | 0,57 | _ | 6,47 | 0,289 |
| *TNFA* (rs1799964) | C C | 1,00 |  |  |  |  |
|  | C T | 0,97 | 0,46 | _ | 2,06 | 0,936 |
| *IL1R (*rs2637988) | A A | 1,00 |  |  |  |  |
|  | A G | 0,96 | 0,53 | _ | 1,72 | 0,879 |
|  | G G | 0,82 | 0,37 | _ | 1,80 | 0,614 |
| *GALNT12* (rs10987898) | G G | 1,00 |  |  |  |  |
|  | G T | 1,36 | 0,76 | _ | 2,44 | 0,303 |
|  | T T | 4,24 | 0,90 | _ | 20,11 | 0,069 |
| *IL4R* (rs1805010) | A A | 1,00 |  |  |  |  |
|  | A G | 0,97 | 0,54 | _ | 1,75 | 0,932 |
|  | G G | 1,41 | 0,68 | _ | 2,93 | 0,352 |
| *LTA* (rs909253) | C C | 1,00 |  |  |  |  |
|  | T C | 0,90 | 0,50 | _ | 1,60 | 0,711 |
|  | T T | 0,96 | 0,41 | _ | 2,23 | 0,921 |
| *IL10RB* (rs1058867) | A A | 1,00 |  |  |  |  |
|  | A G | 0,83 | 0,47 | _ | 1,47 | 0,530 |
|  | G G | 0,71 | 0,31 | _ | 1,62 | 0,416 |
| *IL12A* (rs485497) | A A | 1,00 |  |  |  |  |
|  | A G | 1,66 | 0,93 | _ | 2,95 | 0,084 |
|  | G G | 1,35 | 0,57 | _ | 3,19 | 0,489 |
| *CTLA4* (rs231775) | A A | 1,00 |  |  |  |  |
|  | A G | 1,14 | 0,63 | _ | 2,04 | 0,665 |
|  | G G | 1,90 | 0,81 | _ | 4,46 | 0,139 |
| *IL4RA* (rs1801275) | A A | 1,00 |  |  |  |  |
|  | A G | 1,29 | 0,74 | _ | 2,25 | 0,374 |
|  | G G |  |  |  |  |  |
| *MBL2* (rs5030737) | C C | 1,00 |  |  |  |  |
|  | T C | 0,70 | 0,29 | _ | 1,69 | 0,428 |
| *MBL2* (rs1800450) | A A | 1,00 |  |  |  |  |
|  | G A | 0,87 | 0,46 | _ | 1,67 | 0,682 |
|  | G G | 0,90 | 0,21 | _ | 3,79 | 0,883 |
| *MBL2* (rs1800451) | G G | 1,00 |  |  | - | - |
| *IL10RA* (rs9610) | A A | 1,00 |  |  |  |  |
|  | A G | 1,16 | 0,66 | _ | 2,03 | 0,610 |
|  | G G | 0,97 | 0,41 | _ | 2,30 | 0,941 |
| *IL1B* (rs1143627) | C C | 1,00 |  |  |  |  |
|  | C T | 1,20 | 0,69 | _ | 2,06 | 0,520 |
|  | T T | 1,89 | 0,75 | _ | 4,82 | 0,179 |
| *IL1B* (rs16944) | A A | 1,00 |  |  |  |  |
|  | G A | 0,85 | 0,47 | _ | 1,53 | 0,589 |
|  | G G | 1,79 | 0,71 | _ | 4,51 | 0,216 |
| *IL1B* (rs1143623) | C C | 1,00 |  |  |  |  |
|  | G C | 1,12 | 0,65 | _ | 1,94 | 0,690 |
|  | G G | 1,63 | 0,62 | _ | 4,32 | 0,325 |
| IL10 rs1800871 | C C | 1,00 |  |  |  |  |
|  | T C | 1,13 | 0,57 | _ | 2,23 | 0,732 |
|  | T T | 1,57 | 0,46 | _ | 5,35 | 0,467 |
| IL10 rs1800896 | A A | 1,00 |  |  |  |  |
|  | A G | 0,82 | 0,44 | _ | 1,53 | 0,527 |
|  | G G | 0,88 | 0,45 | _ | 1,74 | 0,720 |
