# Supplementary material for: Inherited Inflammatory Response Genes Are Associated with B-Cell Non-Hodgkin’s Lymphoma Risk and Survival
Source: PLoS One. 2015 Oct 8;10(10):e0139329. doi: 10.1371/journal.pone.0139329 (PMC4598167; doi:10.1371/journal.pone.0139329)
Supplement: S8 Table — (DOCX) [file pone.0139329.s009.docx]

**S8 Table. Gene-gene interactions in relation to overall survival in all patients**

| **SNP** | **Genotype** | **n** | **HR (95% CI)** | **p-value** | **Genotype** | **n** | **HR (95% CI)** | **p-value** | **Genotype** | **n** | **HR (95% CI)** | **p-value** |
| --- | --- | --- | --- | --- | --- | --- | --- | --- | --- | --- | --- | --- |
| ***TNFRSF1B* (rs1061622)** | **TT** |  |  |  | **GT** |  |  |  | **GG** |  |  |  |
| *IL2RA* (rs2104286) |  |  |  |  |  |  |  |  |  |  |  |  |
|  | AA | 110 | 1.00 |  |  | 53 | 1.68 (1.11-2.54) | **0.015** |  | 18 | 1.64 (0.85-3.16) | 0.140 |
|  | GA | 65 | 1.20 (0.80-1.81) | 0.373 |  | 40 | 0.75 (0.44-1.26) | 0.273 |  | 7 | 0.85 (0.31-2.38) | 0.760 |
|  | GG | 16 | 0.14 (0.03-0.57) | **0.006** |  | 10 | 3.11 (1.46-6.61) | **0.003** |  | 2 | - | - |
|  |  |  |  |  |  |  |  |  |  |  |  |  |
| ***MBL2* (rs1800450)** | **GG** |  |  |  | **GA** |  |  |  | **AA** |  |  |  |
| *CTLA4* (rs231775) |  |  |  |  |  |  |  |  |  |  |  |  |
|  | AA | 80 | 1.00 |  |  | 27 | 1.12 (0.64-1.99) | 0.688 |  | 8 | 0.11 (0.01-0.77) | **0.027** |
|  | AG | 122 | 0.88 (0.61-1.28) | 0.510 |  | 28 | 0.78 (0.43-1.41) | 0.410 |  | 4 | 5.13 (1.78-14.77) | **0.002** |
|  | GG | 49 | 0.68 (0.41-1.13) | 0.137 |  | 8 | 2.23 (0.87-5.72) | 0.094 |  | 2 | 3.45 (0.81-14.69) | 0.094 |
|  |  |  |  |  |  |  |  |  |  |  |  |  |
| ***IL12RB1* (rs2305742)** | **AA** |  |  |  | **AC** |  |  |  | **CC** |  |  |  |
| *TNFSF7* (rs16994592) |  |  |  |  |  |  |  |  |  |  |  |  |
|  | TT | 169 | 1.00 |  |  | 83 | 0.92 (0.63-1.34) | 0.665 |  | 19 | 1.89 (1.09-3.29) | **0.024** |
|  | TC | 24 | 1.42 (0.85-2.36) | 0.180 |  | 12 | 0.59 (0.18-1.92) | 0.385 |  | 2 | - | **-** |
|  | CC | 1 | - | - |  | 2 | 19.91 (4.17-95.04) | **<0.001** |  | 0 | - | - |
|  |  |  |  |  |  |  |  |  |  |  |  |  |
| ***IL10RB* (rs1058867)** | **AA** |  |  |  | **AG** |  |  |  | **GG** |  |  |  |
| *TNFA* (rs1800629) |  |  |  |  |  |  |  |  |  |  |  |  |
|  | GG | 79 | 1.00 |  |  | 91 | 1.14 (0.74-1.75) | 0.557 |  | 35 | 1.76 (1.02-3.04) | **0.041** |
|  | AG | 44 | 1.62 (1.01-2.60) | **0.046** |  | 45 | 1.55 (0.93-2.59) | 0.092 |  | 8 | 0.52 (0.12-2.18) | 0.371 |
|  | AA | 10 | 4.99 (2.26-11.00) | **<0.001** |  | 9 | 0.52 (0.16-1.70) | 0.281 |  | 2 | - | - |
|  |  |  |  |  |  |  |  |  |  |  |  |  |
| ***CX3CR1* (rs373379)** | **CC** |  |  |  | **CT** |  |  |  | **TT** |  |  |  |
| *IL12RB1* (rs2305742) |  |  |  |  |  |  |  |  |  |  |  |  |
|  | AA | 114 | 1.00 |  |  | 80 | 0.71 (0.48-1.06) | 0.093 |  | 19 | 2.02 (1.14-3.60) | **0.016** |
|  | AC | 54 | 0.66 (0.41-1.06) | 0.086 |  | 41 | 1.02 (0.62-1.68) | 0.929 |  | 6 | 6.79 (2.63-17.55) | **<0.001** |
|  | CC | 12 | 1.69 (0.84-3.41) | 0.142 |  | 8 | 2.46 (0.97-6.25) | 0.057 |  | 1 | - | - |
|  |  |  |  |  |  |  |  |  |  |  |  |  |
| ***SELE* (rs5361)** | **AA** |  |  |  | **CA** |  |  |  | **CC** |  |  |  |
| *CHI3L1* (rs4950528) |  |  |  |  |  |  |  |  |  |  |  |  |
|  | CC | 175 | 1.00 |  |  | 35 | 1.09 (0.66-1.80) | 0.722 |  | 8 | - | **-** |
|  | CG | 73 | 1.38 (0.97-1.96) | 0.073 |  | 18 | 0.73 (0.38-1.42) | 0.356 |  | 2 | 4.63 (1.10-19.39) | **0.036** |
|  | GG | 7 | 0.61 (0.22-1.68) | 0.334 |  | 4 | 0.46 (0.06-3.30) | 0.437 |  | 0 | - | - |
|  |  |  |  |  |  |  |  |  |  |  |  |  |
| ***IL1B* (rs1143627)** | **TT** |  |  |  | **CT** |  |  |  | **CC** |  |  |  |
| *TNFSF7* (rs16994592) |  |  |  |  |  |  |  |  |  |  |  |  |
|  | TT | 127 | 1.00 |  |  | 115 | 1.54 (1.09-2.18) | **0.015** |  | 30 | 1.20 (0.69-2.08) | 0.529 |
|  | CT | 22 | 1.79 (1.00-3.18) | **0.049** |  | 12 | 1.50 (0.68-3.31) | 0.321 |  | 4 | - | - |
|  | CC | 2 | 28.62 (5.89-139.01) | **<0.001** |  | 0 | - | - |  | 1 | - | - |
|  |  |  |  |  |  |  |  |  |  |  |  |  |
| ***IRF2* (rs3775567)** | **CC** |  |  |  | **CT** |  |  |  | **TT** |  |  |  |
| *MBL2* (rs7096206) |  |  |  |  |  |  |  |  |  |  |  |  |
|  | CC | 185 | 1.00 |  |  | 22 | 1.07 (0.57-2.01) | 0.832 |  | 1 | 5.68 (0.75-42.77) | 0.092 |
|  | CG | 106 | 1.31 (0.95-1.80) | 0.105 |  | 6 | - | - |  | 0 | - | - |
|  | GG | 17 | 1.57 (0.83-2.94) | 0.163 |  | 1 | - | - |  | 0 | - | - |
|  |  |  |  |  |  |  |  |  |  |  |  |  |
| ***IL10* (rs1800896)** | **GG** |  |  |  | **AG** |  |  |  | **AA** |  |  |  |
| *IL4RA* (rs1805010) |  |  |  |  |  |  |  |  |  |  |  |  |
|  | AA | 36 | 1.00 |  |  | 42 | 2.21 (1.18-4.16) | **0.014** |  | 23 | 1.45 (0.66-3.17) | 0.357 |
|  | AG | 42 | 2.48 (1.31-4.69) | **0.005** |  | 84 | 1.57 (0.88-2.83) | 0.130 |  | 42 | 1.40 (0.70-2.79) | 0.345 |
|  | GG | 16 | 3.27 (1.51-7.11) | **0.003** |  | 27 | 1.20 (0.56-2.55) | 0.643 |  | 17 | 3.00 (1.41-6.38) | **0.004** |
