# Supplementary material for: Inherited Inflammatory Response Genes Are Associated with B-Cell Non-Hodgkin’s Lymphoma Risk and Survival
Source: PLoS One. 2015 Oct 8;10(10):e0139329. doi: 10.1371/journal.pone.0139329 (PMC4598167; doi:10.1371/journal.pone.0139329)
Supplement: S9 Table — (DOCX) [file pone.0139329.s010.docx]

**S9 Table.Gene-gene interactions in relation to overall survival in DLBCL**

| **SNP** | **Genotype** | **n** | **HR (95% CI)** | **p-value** | **Genotype** | **n** | **HR (95% CI)** | | **p-value** | | **Genotype** | **n** | | **HR (95% CI)** | | **p-value** |
| --- | --- | --- | --- | --- | --- | --- | --- | --- | --- | --- | --- | --- | --- | --- | --- | --- |
| ***IL4RA* (rs1805010)** | **AA** |  |  |  | **AG** |  |  | |  | | **GG** |  | |  | |  |
| *IL10* (rs1800890) |  |  |  |  |  |  |  | |  | |  |  | |  | |  |
|  | TT | 23 | 1.00 |  |  | 36 | 0.68 (0.33-1.40) | | 0.296 | |  | 14 | | 1.13 (0.52-2.49) | | 0.755 |
|  | TA | 18 | 1.31 (0.63-2.72) | 0.475 |  | 40 | 0.93 (0.48-1.80) | | 0.834 | |  | 7 | | 0.73 (0.30-1.76) | | 0.477 |
|  | AA | 11 | 0.11 (0.02-0.50) | **0.004** |  | 17 | 1.63 (0.76-3.49) | | 0.213 | |  | 6 | | 0.64 (0.19-2.11) | | 0.465 |
|  |  |  |  |  |  |  |  | |  | |  |  | |  | |  |
| ***FCGR2A* (rs1801274)** | **TT** |  |  |  | **TC** |  |  | |  | | **CC** |  | |  | |  |
| *IL1B* (rs419598) |  |  |  |  |  |  |  | |  | |  |  | |  | |  |
|  | GG | 10 | 1.00 |  |  | 27 | 3.31 (1.13-9.71) | | **0.029** | |  | 23 | | 0.78 (0.24-2.52) | | 0.672 |
|  | AG | 18 | 2.71 (0.85-8.64) | 0.091 |  | 50 | 2.18 (0.75-6.35) | | 0.154 | |  | 25 | | 0.82 (0.26-2.58) | | 0.741 |
|  | AA | 4 | 0.36 (0.06-2.00) | 0.241 |  | 17 | 0.91 (0.25-3.28) | | 0.881 | |  | 10 | | 2.39 (0.70-8.19) | | 0.166 |
|  |  |  |  |  |  |  |  | |  | |  |  | |  | |  |
| ***IL10* (rs1800896)** | **GG** |  |  |  | **AG** |  |  | |  | | **AA** |  | |  | |  |
| *IL4RA* (rs1805010) |  |  |  |  |  |  |  | |  | |  |  | |  | |  |
|  | AA | 23 | 1.00 |  |  | 25 | 4.66 (2.04-10.69) | | **<0.001** | |  | 12 | | 2.66 (1.00-7.07) | | 0.050 |
|  | AG | 28 | 4.99 (2.25-11.07) | **<0.001** |  | 49 | 2.73 (1.29-5.80) | | **0.009** | |  | 25 | | 2.24 (0.89-5.60) | | 0.086 |
|  | GG | 9 | 3.11 (1.02-9.47) | **0.046** |  | 20 | 2.10 (0.85-5.16) | | 0.107 | |  | 8 | | 3.50 (1.26-9.74) | | **0.016** |
|  |  |  |  |  |  |  |  | |  | |  |  | |  | |  |
| ***IL10* (rs1800896)** | **GG** |  |  |  | **AG** |  |  | |  | | **AA** |  | |  | |  |
| *IL1RA* (rs419598) |  |  |  |  |  |  |  | |  | |  |  | |  | |  |
|  | TT | 29 | 1.00 |  |  | 33 | 0.45 (0.22-0.91) | | **0.026** | |  | 22 | | 0.94 (0.41-2.17) | | 0.886 |
|  | CT | 16 | 0.38 (0.16-0.90) | **0.027** |  | 34 | 0.87 (0.47-1.64) | | 0.676 | |  | 12 | | 0.91 (0.11-7.42) | | 0.933 |
|  | CC | 3 | - | **-** |  | 5 | 0.95 (0.31-2.94) | | 0.932 | |  | 3 | | - | | - |
|  |  |  |  |  |  |  |  | |  | |  |  | |  | |  |
| ***IL10* (rs1800890)** | **TT** |  |  |  | **TA** |  |  | |  | | **AA** |  | |  | |  |
| *TNFRSF1B* (rs1061622) |  |  |  |  |  |  |  | |  | |  |  | |  | |  |
|  | TT | 44 | 1.00 |  |  | 45 | 0.75 (0.44-1.29) | | 0.300 | |  | 17 | | 0.72 (0.34-1.52) | | 0.384 |
|  | GT | 21 | 0.53 (0.25-1.10) | 0.089 |  | 23 | 2.35 (1.29-4.30) | | **0.005** | |  | 6 | | 0.81 (0.27-2.44) | | 0.714 |
|  | GG | 8 | 1.92 (0.81-4.56) | 0.138 |  | 6 | 0.66 (0.19-2.24) | | 0.503 | |  | 8 | | 0.47 (0.16-1.43) | | 0.185 |
|  |  |  |  |  |  |  |  | |  | |  |  | |  | |  |
| **SNP** | **Genotype** | **n** | **HR (95% CI)** | **p-value** | **Genotype** | **n** | **HR (95% CI)** | | **p-value** | | **Genotype** | **n** | | **HR (95% CI)** | | **p-value** |
| ***TNFRSF1B* (rs1061622)** | **TT** |  |  |  | **GT** |  |  | |  | | **GG** |  | |  | |  |
| *MBL2* (7096206) |  |  |  |  |  |  |  | |  | |  |  | |  | |  |
|  | CC | 60 | 1.00 |  |  | 34 | 1.08 (0.62-1.89) | | 0.783 | |  | 17 | | 2.42 (1.19-4.89) | | **0.014** |
|  | CG | 50 | 1.38 (0.84-2.27) | 0.199 |  | 16 | 4.84 (2.51-9.35) | | **<0.001** | |  | 4 | | 0.40 (0.09-1.74) | | 0.221 |
|  | GG | 6 | 1.51 (0.53-4.34) | 0.441 |  | 3 | 1.23 (0.29-5.32) | | 0.778 | |  | 2 | | 0.75 (0.10-5.69) | | 0.777 |
| ***CX3CR1* (rs373379)** | **CC** |  |  |  | **CT** |  | |  | |  | **TT** | |  |  |  | |
| *IL12RB1* (rs2305742) |  |  |  |  |  |  | |  | |  |  | |  |  |  | |
|  | AA | 67 | 1.00 |  |  | 48 | | 0.59 (0.35-0.97) | | 0.039 |  | | 12 | 2.38 (1.16-4.89) | **0.018** | |
|  | AC | 34 | 0.59 (0.33-1.04) | 0.067 |  | 24 | | 0.98 (0.53-1.80) | | 0.942 |  | | 5 | 4.06 (1.41-11.75) | **0.010** | |
|  | CC | 7 | 1.93 (0.75-4.98) | 0.174 |  | 5 | | 2.42 (0.84-6.96) | | 0.100 |  | | 1 | 0.28 (0.04-2.13) | 0.219 | |
|  |  |  |  |  |  |  | |  | |  |  | |  |  |  | |
| ***IL2RA* (rs2104286)** | **AA** |  |  |  | **GA** |  | |  | |  | **GG** | |  |  |  | |
| *BAFF* (rs9514828) |  |  |  |  |  |  | |  | |  |  | |  |  |  | |
|  | AA | 28 | 1.00 |  |  | 24 | | 1.78 (0.91-3.48) | | 0.095 |  | | 2 | - | - | |
|  | GA | 61 | 1.23 (0.67-2.25) | 0.577 |  | 25 | | 0.76 (0.37-1.56) | | 0.451 |  | | 9 | 0.71 (0.20-2.51) | 0.600 | |
|  | GG | 28 | 1.73 (0.88-3.41) | **0.024** |  | 16 | | 0.82 (0.35-1.94) | | 0.652 |  | | 4 | 1.72 (0.48-6.20) | 0.407 | |
|  |  |  |  |  |  |  | |  | |  |  | |  |  |  | |
| ***IL10* (rs1800872)** | **CC** |  |  |  | **AC** |  | |  | |  | **AA** | |  |  |  | |
| *IL2* (rs2069762) |  |  |  |  |  |  | |  | |  |  | |  |  |  | |
|  | TT | 51 | 1.00 |  |  | 28 | | 0.99 (0.51-1.92) | | 0.984 |  | | 2 | 32.03 (6.38-160.75) | **<0.001** | |
|  | GT | 31 | 1.19 (0.65-2.19) | 0.577 |  | 20 | | 1.04 (0.53-2.01) | | 0.913 |  | | 6 | 0.15 (0.02-1.14) | 0.067 | |
|  | GG | 15 | 2.40 (1.12-5.13) | **0.024** |  | 4 | | 0.61 (0.08-4.56) | | 0.626 |  | | 1 | 4.65 (0.60-36.27) | 0.143 | |
|  |  |  |  |  |  |  | |  | |  |  | |  |  |  | |
| ***IL10* (rs1800871)** | **CC** |  |  |  | **CT** |  | |  | |  | **TT** | |  |  |  | |
| *IL4RA* (rs1805010) |  |  |  |  |  |  | |  | |  |  | |  |  |  | |
|  | AA | 33 | 1.00 |  |  | 14 | | 2.83 (1.31-6.08) | | **0.008** |  | | 5 | 1.32 (0.35-4.93) | 0.679 | |
|  | AG | 52 | 2.40 (1.32-4.39) | **0.004** |  | 29 | | 1.07 (0.52-2.22) | | 0.851 |  | | 8 | 0.69 (0.16-3.04) | 0.623 | |
|  | GG | 22 | 1.24 (0.58-2.66) | 0.575 |  | 5 | | 2.95 (0.96-9.09) | | 0.060 |  | | 2 | 3.70 (0.80-17.14) | 0.095 | |
