# Supplementary material for: Inherited Inflammatory Response Genes Are Associated with B-Cell Non-Hodgkin’s Lymphoma Risk and Survival
Source: PLoS One. 2015 Oct 8;10(10):e0139329. doi: 10.1371/journal.pone.0139329 (PMC4598167; doi:10.1371/journal.pone.0139329)
Supplement: S10 Table — (DOCX) [file pone.0139329.s011.docx]

**S10 Table. Gene-gene interactions in relation to overall survival in FL**

| **SNP** | **Genotype** | **n** | **HR (95% CI)** | **p-value** | **Genotype** | **n** | **HR (95% CI)** | **p-value** | **Genotype** | **n** | **HR (95% CI)** | **p-value** |
| --- | --- | --- | --- | --- | --- | --- | --- | --- | --- | --- | --- | --- |
| ***IL1RA* (rs2637988)** | **AA** |  |  |  | **AG** |  |  |  | **GG** |  |  |  |
| *IL4* (rs2243248) |  |  |  |  |  |  |  |  |  |  |  |  |
|  | TT | 47 | 1.00 |  |  | 53 | 0.63 (0.33-1.19) | 0.152 |  | 19 | 0.76 (0.33-1.75) | 0.512 |
|  | GT | 4 | - | - |  | 6 | 3.01 (1.10-8.26) | **0.032** |  | 3 | 0.45 (0.10-2.12) | 0.315 |
|  | GG | 0 | - | - |  | 1 | 4.23 (0.52-34.40) | 0.178 |  | 6 | - | - |
|  |  |  |  |  |  |  |  |  |  |  |  |  |
| ***TNFRSF1B* (rs1061622)** | **TT** |  |  |  | **GT** |  |  |  | **GG** |  |  |  |
| *IL2RA* (rs2104286) |  |  |  |  |  |  |  |  |  |  |  |  |
|  | AA | 46 | 1.00 |  |  | 22 | 1.43 (0.67-3.06) | 0.359 |  | 2 | 1.61 (0.20-12.81) | 0.653 |
|  | GA | 20 | 0.93 (0.39-2.20) | 0.872 |  | 24 | 0.82 (0.38-1.75) | 0.604 |  | 2 | - | - |
|  | GG | 9 | - | - |  | 4 | 5.28 (1.43-19.48) | **0.013** |  | 0 | - | - |
|  |  |  |  |  |  |  |  |  |  |  |  |  |
