# Supplementary material for: Inherited Inflammatory Response Genes Are Associated with B-Cell Non-Hodgkin’s Lymphoma Risk and Survival
Source: PLoS One. 2015 Oct 8;10(10):e0139329. doi: 10.1371/journal.pone.0139329 (PMC4598167; doi:10.1371/journal.pone.0139329)
Supplement: S1 Text — (DOCX) [file pone.0139329.s012.docx]

**S12 Text**

**Ethics statement and patient data**

### Patients

The current study included genotyping of 355 patients with available formalin-fixed paraffin-embedded (FFPE) bone-marrow aspirate diagnosed at Aalborg University Hospital in the period 1999-2005. Of the 355 present B-NHL cases, 216 had DLBCL and 139 had FL. The patients included were Caucasian and described not to have bone marrow (BM) involvement at the time of diagnosis. Clinical data was abstracted from the Danish lymphoma database (LYFO) for 310 patients. Forty five patients were not registered in LYFO or did not have a complete dataset. For 32 patients, clinical data was abstracted from medical records. The remaining 13 patients died within a few days after being diagnosed with lymphoma, this group did not provide sufficient clinical data and were excluded from outcome analysisAll lymphomas were classified according to the World Health Organization (WHO) classification [1]. Vital status was abstracted from the Danish death registry. From LYFO or medical records we obtained follow-up information including type of treatment and IPI score. All patients were treated with standard CHOP based regimens. Research protocols were approved by the local scientific ethics committee of the North Denmark Region, approval **N-20100059**.

### Healthy controls

The healthy controls consisted of 307 healthy blood donors from the Aalborg University Hospital Blood Bank, Aalborg, Denmark. All donors reported Danish ethnicity and were therefore considered Caucasian. The control group was previously described in [2] and we randomly included 307 donors for the genotyping study. The donors routinely sign a statement declaring that they do not take any medication and are not under investigation for any disorder. All donors were clinically healthy at the time of blood drawing. The samples were handled anonymously and only registered with ethnicity, age and sex. All donors gave consent to the blood being used for this purpose. Research protocols were approved by the local scientific ethics committee of the North Denmark Region, approval **N-20090018**.

**Purification of DNA**

Formalin-fixed paraffin-embedded (FFPE) bone-marrow aspirate was obtained from 355 included patients. From the FFPE, ten 5 μm sections were cut for molecular analysis. The microtome was carefully cleansed before processing material from a new patient. Paraffin was removed by heating. DNA was extracted from the tissue samples using an automated Maxwell® 16 low elution volume tissue DNA Purification Kit (Promega, Madison WI, USA). DNA concentration and quality was evaluated using a Nanodrop 8000 UV-Vis Spectrophotometer (Thermo Scientific, Wilmington DE, USA). Ethylenediaminetetraacetic acid (EDTA) stabilised whole blood was collected from the blood donors. DNA were extracted from 200 μl volumes of whole blood and were eluted into a total volume of 50 μl ATE buffer using the QIAsymphony DNA Mini Kit (QIAGEN, Germany) on the Qiasymphony SP platform according to the manufacturer’s instructions. All DNA samples were diluted to a final concentration of 50 ng/μl in 96-well plates.

### Genotyping


The included SNPs were selected from the literature according to their proposed role in hematological malignancies or by their functional properties in inflammation and regulation of the immune system. Genotyping of the 50 SNPs was performed using a TaqMan OpenArray genotyping system from Applied Biosystems (ABI, Foster City, CA, USA). The OpenArray system is a high-throughput, highly automated and relatively low-cost (per assay) system that allow testing of many SNPs in multiple individuals in parallel. 7 SNPs was typed using custom-designed assays and 43 SNPs was typed using predesigned TaqMan SNP assays (see **supplementary table 1** for detailed assay information). OpenArray plates were manufactured by Applied Biosystems (ABI, Foster City, CA, USA). A nontemplate control (NTC) was introduced within each set of assays. TaqMan OpenArray master mix (ABI, Foster City, CA, USA) was used in this study according to the manufacture’s protocol. Samples were loaded into OpenArray plates using the OpenArray NT Autoloader and cycled using GeneAmp 9700 thermal cycler with PCR conditions according to the manufacturer’s protocol (ABI, Foster City, CA, USA). The arrays were read using the OpenArray NT Imager and the allele calls and scatter plots were generated with the Biotrove OpenArray SNP Genotyping Analysis Software package version 1.0.3. All data is analyzed with the the Biotrove OpenArray SNP Genotyping Analysis Software which is a SNP genotyping data analysis tool to analyze raw data from genotyping experiments performed on the Open Array system (Applied Biosystem). The default threshold for the Quality value was set to 0.95. The score is an estimate of how closely a given data point belongs to those underlying models of angle and amplitude distributions for each genotype cluster. The call rate was >80% for all genotypes tested.1. Jaffe ES. The 2008 WHO classification of lymphomas: implications for clinical practice and translational research. Hematology Am Soc Hematol Educ Program. 2009; 523–31. doi:10.1182/asheducation-2009.1.523

2. Nielsen KR, Steffensen R, Boegsted M, Baech J, Lundbye-Christensen S, Hetland ML, et al. Promoter polymorphisms in the chitinase 3-like 1 gene influence the serum concentration of YKL-40 in Danish patients with rheumatoid arthritis and in healthy subjects. Arthritis Res Ther. 2011;13: R109. doi:10.1186/ar3391
